# Population genomics, life‐history tactics, and mixed‐stock subsistence fisheries in the northernmost American Atlantic salmon populations

**DOI:** 10.1111/eva.13654

**Published:** 2024-02-22

**Authors:** Alexandre Carbonneau, Julien April, Eric Normandeau, Anne‐Laure Ferchaud, Véronique Nadeau, Louis Bernatchez

**Affiliations:** ^1^ Département de Biologie, Institut de Biologie Intégrative et des Systèmes (IBIS) Université Laval Québec Quebec Canada; ^2^ Ministère de l'Environnement, de la Lutte contre les changements climatiques, de la Faune et des Parcs (MELCCFP) Québec Quebec Canada; ^3^ Parks Canada, Office of the Chief Ecosystem Scientist, Protected Areas Establishment Québec Quebec Canada

**Keywords:** Atlantic salmon, fishery conservation and management, life‐history tactics, mixed‐stock fishery, population genomics, subsistence harvesting

## Abstract

While Atlantic salmon (*Salmo salar*) of the northernmost American populations is alimentary, economically, and culturally important for Ungava Inuit communities (Nunavik, Canada) and might play a key role in the persistence of the species in a global warming context, many mysteries remain about those remote and atypical populations. Thus, our first aim was to document the genomic structure of the Nunavik populations. The second objective was to determine whether salmon only migrating to the estuary without reaching the sea, apparently unique to those populations, represent distinct populations from the typical anadromous salmons and subsequently explore the genetic basis of migratory life‐history tactics in the species. Finally, the third goal was to quantify the contribution of each genetically distinct population and life‐history tactic in the mixed‐stock subsistence fishery of the Koksoak R. estuary. We used Genotyping‐by‐Sequencing to genotype 14,061 single nucleotide polymorphisms in the genome of 248 individuals from 8 source populations and 280 individuals from the Koksoak estuary mixed‐stock fishery. Life‐history tactics were identified by a visual assessment of scales. Results show a hierarchical structure mainly influenced by isolation‐by‐distance with 7 populations out of the 8 studied rivers. While no obvious structure was detected between marine and estuarine salmon within the population, we have identified genomic regions putatively associated with those migration tactics. Finally, all salmon captured in the Koksoak estuary originated from the Koksoak drainage and mostly from 2 tributaries, but no inter‐annual variation in the contribution of these tributaries was found. Our results indicate, however, that both marine and estuarine salmon contribute substantially to estuarine fisheries and that there is inter‐annual variation in this contribution. These findings provide crucial information for the conservation of salmon populations in a rapidly changing ecosystem, as well as for fishery management to improve the food security of Inuit communities.

## 1 INTRODUCTION

One of the main goals of conservation biology is to maintain evolutionary processes and the ecological viability of populations that constitute a species (Moritz, 1999). Since the components of the species' genetic or ecological diversity that will be important in the future are unknown, it is necessary to maintain as many genetically distinct populations as possible (Funk et al., 2012; Palsbøll et al., 2007; Waples, 1995). In a species conservation and management context, one of the first important steps is to identify and group populations into conservation units (CUs) based on their similarities so that managers benefit from a clear framework in which to apply common management actions across similar populations (Funk et al., 2012). There are several types of CUs, but they usually consist of groups of populations that are demographically distinct (e.g. management units (MUs); Moritz, 1994; Palsbøll et al., 2007) or that represent substantially divergent entities over evolutionary timescales (e.g. evolutionarily significant units; ESU; Allendorf et al., 2013; Fraser & Bernatchez, 2001).

The implementation of CUs requires a considerable amount of knowledge about the genetic, ecological, and geographic structure of populations, as well as about the biology of the species in general. However, such knowledge is still lacking for many species in some parts of their range of distribution, especially in remote areas. This is the case for many fish species that support subsistence fisheries in Northern Canada, which received much less scientific attention than their southern counterparts, which impedes informed management decisions (Poesch et al., 2016) and creates inequity for indigenous communities living at these latitudes. This is particularly true near the poles, where past temperature records show that global warming is occurring faster than in the rest of the world, and projections suggest that this trend will likely be maintained (IPCC, 2021).

Many Inuit communities across northern Canada rely heavily on natural resources for food security, social cohesion and integration, and the transmission of cultural knowledge and values (Power, 2008). Throughout Nunangat (all Inuit territories), more than half of Inuit endure food insecurity (Arriagada, 2017), mostly because of a combination of high prices in grocery stores and poverty levels (Council of Canadian Academies, 2014). Thus, it is especially crucial to improve scientific knowledge about the species harvested by northern indigenous communities in order to improve their management and limit food insecurity in the long term.

Atlantic salmon (*Salmo salar*), hereafter referred to as “salmon”, is a socio‐economically important species in the Ungava Bay region (Nunavik, Canada). Although Arctic char (*Salvelinus alpinus*) is by far the most harvested fish in Nunavik (Blanchet & Rochette, 2008), its virtual absence in the Koksoak River has led inhabitants of Kuujjuaq, Nunavik's most populous village, to rely more heavily on salmon. As a result, the fishery occurring in the Koksoak R. estuary is likely the most important subsistence salmon fishery in Nunavik. As it is well known that salmon can be captured in an estuary different from their river of origin (Bradbury et al., 2015; Colombani et al., 1998; Gauthier‐Ouellet et al., 2009), this subsistence fishery is presumably targeting salmon from different populations that are inside and/or outside the Koksoak system (mixed‐stock fishery).

**FIGURE 1:**
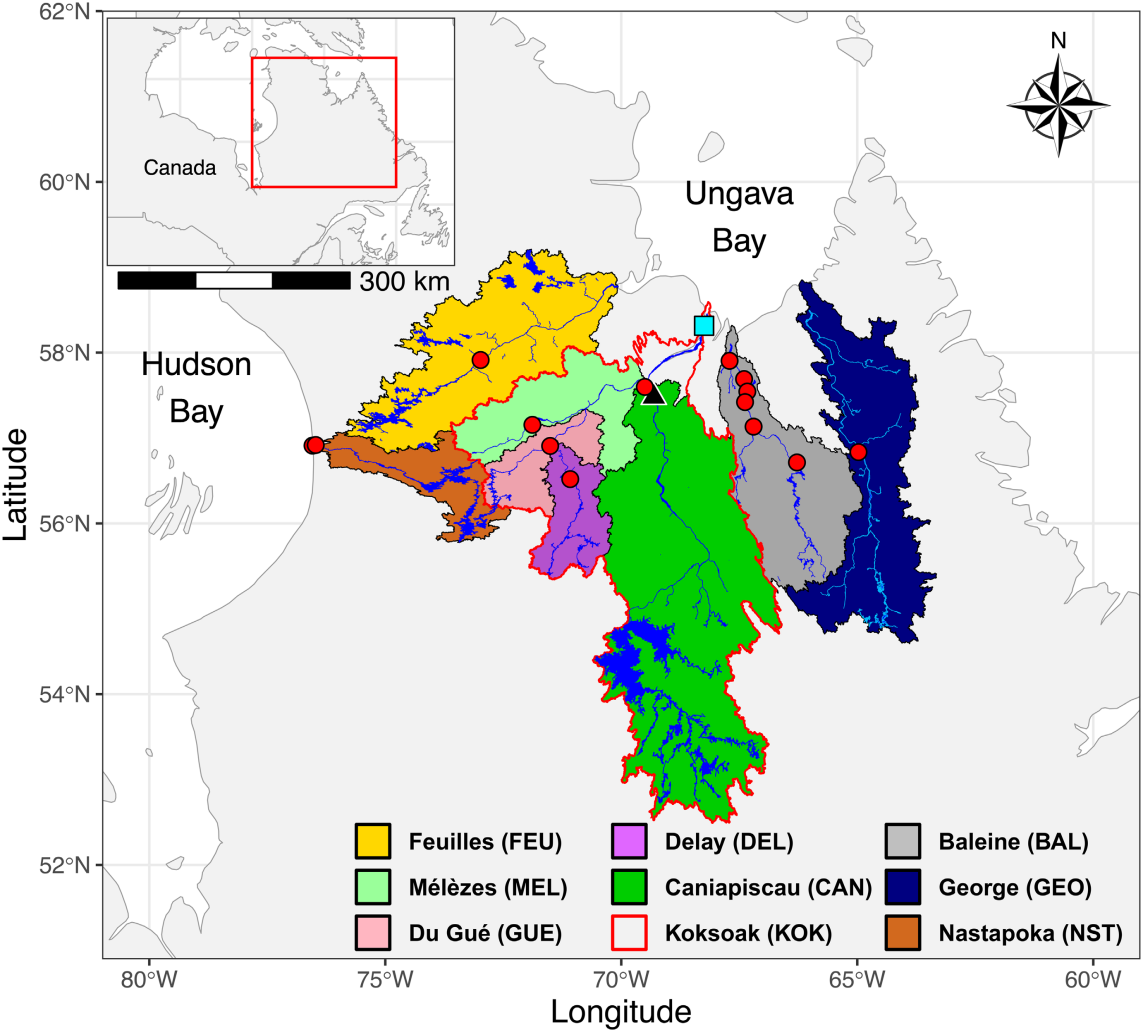
Location of sampling sites (red dots indicate the source populations and the light blue square the mixed stock) in Nunavik salmon rivers. The black triangle represents Limestone Falls, an impassible obstacle for upstream migration.

From a biological perspective, Nunavik salmon populations, namely all Quebec salmon rivers north of the 55th parallel, are exceptional for a few reasons. First, in addition to being the most northerly representatives of the species in North America (MacCrimmon & Gots, 1979), they experience reduced genetic connectivity with more southern populations (Dionne et al., 2008). Furthermore, Ungava Bay salmon, which we describe as all salmon from rivers flowing into Ungava Bay (Feuilles, Koksoak, Baleine, and George; Figure 1), are expected to have developed specific adaptations to their marginal environment. Additionally, scale analyses from a few studies that have focused specifically on salmon from the Koksoak R., which is divided into four different rivers (Mélèzes, Du Gué, Delay, and Caniapiscau; Figure 1), have revealed that this system hosts salmon that exhibit three different migratory life‐history tactics: marine, landlocked, and estuarine (Côté et al., 1984; Power, 1981; Robitaille et al., 1982, 1986). Marine salmon are very similar to the typical anadromous southern counterpart of the species, meaning they migrate to the sea for feeding, sometimes as far as the coast of Greenland (Bradbury et al., 2016), and come back into rivers to breed. Landlocked salmon remain in freshwater for their entire life and are mostly isolated in Caniapiscau R. upstream of Limestone Falls, an obstacle unable to be surmounted (Figure 1), and in a few headwater lakes. Estuarine salmon, which have only been described in the Koksoak R. and thus appear to be unique to the Koksoak R. or Ungava Bay populations, migrate only in their river's estuary and never reach the ocean. This diversity of migration patterns may have helped these northern populations to survive despite the harsh environmental conditions of the north (Robitaille et al., 1986). West of the Ungava Bay in Nunavik, a salmon population, with landlocked individuals and others that seem to be anadromous, has been described in the Hudson Bay and is geographically isolated in the Nastapoka R. (Figure 1). The colonization route to this river and the potential for anadromous salmon to complete their life cycle in this system are not clear (April et al., 2023; Le Jeune & Legendre, 1968; Morin, 1991). Indeed, the resident form has been documented upstream of the 35‐m‐high Nastapoka Falls, but individuals have also been caught in Hudson Bay and in the 1000 m or so of available river habitat between the sea and the Nastapoka Falls (April et al., 2023; Le Jeune & Legendre, 1968; Morin, 1991). Finally, the northernmost parts of the distribution range of Atlantic salmon will very likely become of key importance for the future of the species in a global warming context (Bilous & Dunmall, 2020; Chittenden et al., 2013; Todd et al., 2011).

Despite its biological and socio‐economic value, very little is known about Nunavik's salmon, which complicates its management and reduces its sustainability, contributing to the food insecurity issue. To our knowledge, only three studies have addressed the population structure of Ungava Bay salmon on a subset of rivers draining into this system, one using 13 microsatellite markers (Dionne et al., 2008) and the other two using single nucleotide polymorphisms (SNPs) (Bourret et al., 2013: 3118 SNPs; Moore et al., 2014: 3192 SNPs). These studies showed that three of the described rivers in Ungava Bay (Feuilles, Koksoak, and George) are genetically different from southern populations and from each other. However, these studies were conducted at a large spatial scale (throughout Quebec and across the entire North American species distribution) and did not aim at assessing the fine‐scale patterns across all salmon rivers in Nunavik. Also, these studies did not provide any scientific information pertaining to the genetic basis of life‐history tactic variation in the Koksoak R.

In an effort to improve applied scientific knowledge of Nunavik salmon and contribute to informed management decisions, the present study addresses three main objectives. The first is to document fine‐scale genomic population structure using many more markers compared to previous studies (Bourret et al., 2013; Dionne et al., 2008; Moore et al., 2014), including for the first time all legally described Nunavik salmon rivers and tributaries (Quebec Fishery Regulations, 1990 (SOR/90‐214)), as well as Nastapoka R. in Hudson Bay. The second objective is to identify whether marine and estuarine Koksoak R.'s salmon represent genetically distinct populations and subsequently explore the genetic basis of migratory life‐history tactics in the species. Previous studies suggest that the development of life‐history tactics in salmonids depends on the action of many genes (Dodson et al., 2013; Nichols et al., 2008; Reid et al., 2005) and that the genetic basis of migration is likely indirect and mediated by physiological traits such as growth rate (Kelson et al., 2020). We can thus expect to observe a polygenic signal with several genes across the genome that are likely associated with life‐history tactics. The third goal is to quantify the contribution of each genetically distinct population, whether they are within or outside the Koksoak R., and each life‐history tactic to the mixed‐stock subsistence fishery in the Koksoak R. estuary. Since estuarine salmon spend almost all summer in this environment, and given that fishing also occurs during the summer (peak season in August), we expected to find a greater proportion of estuarine salmon compared to their marine counterparts in the subsistence fishery. This study is part of the Fostering Indigenous Small‐scale Fisheries for Health, Economy, and Food Security (FISHES) project (https://fishes‐project.ibis.ulaval.ca/), which aims at improving genomic knowledge of 7 species (*Salvelinus alpinus*, *Sander vitreus*, *Salvelinus namaycush*, *Salvelinus fontinalis*, *Coregonus clupeaformis*, *Coregonus artedi*, and *Salmo salar*) harvested by indigenous nations (Inuit, Cree, and Dene) across Northern Canada in order to foster collaborative management of these species and ultimately enhance food security. Understanding both the genetic variation underlying the observed migratory tactics and the stock composition in salmon in Ungava Bay will provide insight on how the life‐history variation helps the species to cope with environmental changes (Robitaille et al., 1986) and will consequently facilitate more accurate and informed decisions in terms of fishing periods and quotas.

## 2 MATERIALS AND METHODS

### 2.1 Sampling

Sampling was carried out in isolated sections of rivers, distant from other river connections, to ensure the purest representation of each genetically distinct population. This approach aimed to ensure that each sample accurately reflected its population, optimizing the data for identifying the origins of fish—namely source populations—in mixed‐stock fisheries. A total of 358 individuals were captured from the rivers Feuilles (FEU), Mélèzes (MEL), Du Gué (GUE), Delay (DEL), Caniapiscau (CAN), Baleine (BAL), George (GEO), and Nastapoka (NST) (Figure 1). The vast majority of sampled salmon were returning adults caught by fly fishing by sport anglers (FEU, DEL, GEO, and 17 fish in CAN), or caught in counting fences (MEL and GUE) or using gillnets (NST) by the Ministère de l'Environnement, de la Lutte contre les Changements Climatiques, de la Faune et des Parcs (MELCCFP). The remaining salmon from CAN (*n* = 10) and BAL rivers were respectively caught using gillnets (local indigenous fishers) and electrofishing (MELCCFP, which aimed for fry and parr). Then, sampling of 296 individuals from the mixed‐stock fisheries was conducted over 2 years in the Koksoak (KOK) estuarine subsistence gillnets fisheries. Sampling of the mixed stock was carried out by Inuit fishers from the community of Kuujjuaq in order to be as representative as possible of this fishery, and samples were collected at different times and different locations within the estuary.

The adipose fin was sampled from each fish and preserved in 95% ethanol or RNAlater. We used intercirculi spacing patterns of scales to evaluate the migratory life‐history tactics of individuals caught in source rivers (MEL, GUE, DEL, and CAN) and within the KOK estuary. We sampled fish scales from the fish flank on either side of the lateral line at the level of an imaginary line between the back of the dorsal fin and the front of the anal fin, which were stored dry in individual envelopes.

### 2.2 DNA extraction, Genotyping‐by‐Sequencing (GBS) library preparation, sequencing, and SNP calling

Genomic DNA was extracted using the NucleoMag 96 Tissue kit (Macherey‐Nagel) and the epMotion automated liquid handling system (Eppendorf). 1% agarose gel electrophoresis was used to assess the quality of DNA, and then the quantity and quality were evaluated by a NanoDrop 2000 spectrophotometer (Thermo Scientific). A total of 92 (*n* = 84 from source populations and *n* = 8 from mixed‐stock fisheries) individuals were removed after these steps because of a lack of DNA quality or quantity. 10 μL of DNA was then normalized to 10 ng/μL based on concentrations measured using the AccuClear Ultra High‐Sensitivity dsDNA Quantification Kit (Biotium; SPARK, TECAN) to maximize the accuracy of DNA quantification.

All of the 562 remaining individuals from all source populations and the mixed‐stock fishery were randomly spread over several plates to avoid a possible batch effect. Genotyping‐by‐Sequencing (GBS) libraries were prepared using a modified version of the protocol described by Abed et al. (2019), using the restriction enzymes *PstI* and *MspI*. Sequencing was done at the Plateforme d'Analyses Génomiques (PAG) of IBIS, Laval University, on an Ion Torrent sequencer using the Ion 550 chips, obtaining a mean of 4.62 M single reads (±0.9) per fish. Finally, the DNA volume of each sample was adjusted after sequencing the first batch of chips to improve the uniformity of coverage among samples.

Data preparation, genotyping, and filtration were then done using STACKS v2.55 (Catchen et al., 2013) and stacks_workflow v2.5.4 (https://github.com/enormandeau/stacks_workflow). Briefly, cutadapt v1.18 (‐e 0.2 ‐m 50) was used to trim reads for quality. Samples were demultiplexed with process_radtags v2.62 (‐c ‐r ‐t 120 ‐q ‐s 0 ‐‐barcode_dist_1 2 ‐E phred33 ‐‐renz_1 pstI ‐‐renz_2 mspI). We then aligned the cleaned reads against the reference genome (Ssal_Brian_v1.0, NCBI Genbank accession GCA_923944775.1) using bwa v0.7.17‐r1188 (‐k 19 ‐c 500 ‐O 0,0 ‐E 2,2 ‐T 0) and samtools v1.8 (‐Sb ‐q 1 ‐F 4 ‐F 256 ‐F 2048), followed by gstacks (‐‐max‐clipped) and population (‐p 2 ‐r 0.6 ‐‐ordered‐export ‐‐fasta‐loci ‐‐vcf) modules of the stacks_workflow pipeline. It should be noted that the reference genome used in the analyses is a preliminary version of GCA_923944775.1 that included a fusion between chromosomes ssa08 and ssa29 (Laurie Lecompte personal communication), but positions were subsequently corrected to remove it. Since this fusion is a well‐documented polymorphism present in North American salmon (Lehnert et al., 2019; Wellband et al., 2019), we retained the analyses performed with this preliminary reference genome that included the fusion.

After these STACKS steps, we filtered the SNPs as follows. We first filtered the SNPs so that all genotypes had a minimum coverage of 3×, and we retained SNPs for which all the sample groups had at most 40% of missing data and for which at least 3 samples had the rare allele (05_filter_vcf_fast.py, params: 3 60 0 3). Using this dataset, samples with more than 5% missing data (*n* = 24) were removed from the original populations.snps.vcf file. This new VCF, containing only the highest quality samples, was filtered again so that all genotypes had a minimum coverage of 3× and only SNPs for which all the sample groups had at most 50% missing data and for which at least 5 samples had a copy of the rare allele were kept (05_filter_vcf_fast.py, params: 3 50 0 5). The resulting median proportion of missing data was below 5% for all of the 8 source populations and mixed‐stock individuals, which was considered the 9th population for these filtering steps. Then, we evaluated relatedness using vcftools (‐‐relatedness) with a threshold of 0.9 for each pair of samples and removed 8 individuals to retain only one representative of these possible kin pairs. We also looked at the distribution of heterozygosity among the sample groups and removed 2 individuals with high heterozygosity (>0.3) compared to others (mean = 0.201). We created a new VCF file, this time excluding all the unwanted samples, and proceeded to filter this VCF again (05_filter_vcf_fast.py, params: 3 50 0 5) with the same parameters. Using a modified HD plot approach based on McKinney et al. (2017) and implemented in stacks_workflow, we removed SNPs that displayed signs of paralogy or over‐merging (scripts 08, 09, and 10 from stacks_workflow). Keeping only canonical SNPs, referred to as singletons, we then removed SNPs that were in high linkage disequilibrium within 100 k bp, using a threshold of 0.5. In these cases, we kept only the first of the linked SNPs. We also performed an identity by missingness (IBM) using PLINK (Purcell et al., 2007) and found no cluster created by missing data.

### 2.3 Population structure analyses

Population genetic structure was documented using three different approaches. First, a principal components analysis (PCA) was performed on the genotypes using the R package *ade4* v1.7‐15 (Dray & Dufour, 2007). Second, we estimated ancestry with the maximum likelihood method implemented in the ADMIXTURE software (Alexander et al., 2009) for 1–12 genetic clusters (K). The retained K was selected based on the lowest value of the cross‐validation error. The analysis was repeated with a genetic cluster range of 1–5 within each of the clusters defined by ADMIXTURE. Third, pairwise *F*_ST_ among sampling rivers was assessed with the R package *StAMPP* v1.6.1 (Pembleton et al., 2013) using 1000 bootstraps and a 95% confident interval.

Isolation‐by‐distance (IBD) was tested by considering the relationship between linear *F*_ST_ (*F*_ST_/[1−*F*_ST_]) (Rousset, 1997) and the geographical distances between sampling sites in a simple linear model. Pairwise geographic distances were calculated with QGIS v3.16.1 (QGIS Development Team, 2021) as the minimum distance that salmon must travel from one site to another. Four models were then created, considering: (1) all Ungava Bay's rivers (meaning without NST R, since it is clearly isolated given the absence of connections between Ungava Bay's rivers and Hudson Bay); (2) all Ungava Bay's rivers except CAN R., which presents a particular dynamic due to a unidirectional gene flow from an isolated population; and (3 and 4) a null model (Y~0) for each of these two models. Model selection based on the Akaike information criterion that corrects for sample size (AICc) was then performed using the R package *AICcmodavg* v2.3‐1 (Mazerolle, 2017), and the model with the lowest AICc value was retained.

Directional connectivity between putative source populations was then estimated with the *divMigrate* function (using the *G*_ST_ statistic (Nei, 1973) and 1000 iterations) implemented in the R package *diveRsity* v1.9.90 (Keenan et al., 2013) following the method described by Sundqvist et al. (2016). Briefly, this method consists of creating a hypothetical pool of migrants between two populations and then determining the genetic differentiation (Jost's D or *G*_ST_) between the pool of migrants and each of the two populations. The levels of differentiation thereby obtained can then be used to estimate the relative level of directional gene flow (a value relative to other pairs of populations, not an absolute rate of migration/gene flow value) between these two populations, the one with the lower differentiation value with the migrant pool being considered the population of origin, or where migrants come from. Although the function has been designated for microsatellite markers, it has been shown that *divMigrate* can handle SNPs well (e.g. Garrison et al., 2021; Manuzzi et al., 2019; Woodings et al., 2018).

### 2.4 Genetic diversity within sampling sites

The expected (He) and observed (Ho) heterozygosity, as well as *F*_IS_, were calculated per individual using VCFtools v0.1.13 (Danecek et al., 2011) and then averaged by river. The effective population size (*N*_e_) of each putative source population was estimated with NeEstimator v2.1 (Do et al., 2014) using the linkage disequilibrium method with markers having a minor allele frequency (MAF) greater than 0.05 and applying a correction to remove linked loci across chromosomes (Waples et al., 2016).

### 2.5 Genetic basis of life‐history tactics

The identification of the migratory tactics was done by MELCCFP using a stereo microscope to visualize scale intercirculi spacing according to the methods of Côté et al. (1984) and Robitaille et al. (1986) (see Data S1 for more details), which is more cost‐effective and less time‐consuming, while being as effective and relevant as microchemical analyses of otoliths (Brûlé, 2022). This scale reading method was applied to all individuals caught within the Koksoak R. (estuarine fishery as well as source rivers: MEL, GUE, DEL, CAN), but not to those caught within FEU, BAL, GEO, and NST. Individuals from the latest 4 rivers were thus removed from the following analysis. Fish from CAN R. were also removed due to its strong differentiation from other rivers, which would have been likely to create structure‐related noise between rivers rather than between life‐history tactics. In addition, individuals that were caught in the estuary (mixed stock) and that were assigned to the 3 remaining rivers (MEL, GUE, and DEL) were included in the dataset (see section on mixed‐stock assignment for details), increasing the sample size from 99 individuals to 304, with a total (individuals captured in river and in estuary) of 32 individuals from MEL R. (*n*_estuarine_ = 10, *n*_marine_ = 22), 99 from GUE R. (*n*_estuarine_ = 70, *n*_marine_ = 29), and 173 from DEL R. (*n*_estuarine_ = 108, *n*_marine_ = 65) (Table S1).

In order to understand how genetic diversity is partitioned among life‐history tactics or rivers, two analyses of molecular variance (AMOVA) were performed for the MEL, GUE, and DEL rivers using the R package *poppr* v2.9.3 (Kamvar et al., 2014). In both cases, the dependent variable was a genotype matrix (genind format), and the independent variables were river and migratory life‐history tactics. In one model, river and migratory life‐history tactic were respectively considered as the primary and secondary groups, and in the second model, the order of these variables was inverted so that migratory life‐history tactic and river were considered as the primary and secondary group, respectively. The goal was to investigate the proportion of variation partitioned between rivers versus between migratory life‐history tactics within rivers (model 1) and vice versa (between life‐history tactics versus between rivers within life‐history tactics; model 2). A *p* value was then obtained using a randomization test (*randtest*) using the R package *ade4* v1.7–15, with 1000 iterations, and a PCA was also performed to visualize this relationship.

SNPs that were significantly associated with life‐history tactics were then identified using outlier detection analysis as implemented in the R package *pcadapt* v4.3.3 (Privé et al., 2020). The number of PCs (named K in the function) was first set to *K* = 20, and the visual scree plot method was used to determine the value of K that explained the most variance. Next, the *p* values were transformed into *q* values using the R package *qvalue* v2.28.0 (Storey et al., 2022), with a threshold of 0.01. Finally, we annotated the genome assembly using the pipeline GAWN v0.3.5 (https://github.com/enormandeau/gawn) and then identified all genes within 20 kb surrounding outlier SNPs in order to test for functional enrichment of gene ontology (GO) biological process terms using R package *topGO* v2.50.0 (Alexa & Rahnenfuhrer, 2022). We used the “weight01” algorithm and the Kolmogorov–Smirnov (KS) statistic to perform enrichment tests.

### 2.6 Mixed‐stock analysis

Once population structure was characterized, the performance of the dataset (*n* = 248, with 14,061 SNPs) in assigning individuals to their population of origin was evaluated using an assignment test via Monte–Carlo cross‐validation with 100 iterations, implemented in the R package *assignPOP* v1.2.4 (Chen et al., 2020). For each iteration, a given proportion of individuals is randomly sampled in each population of the dataset, which generates the training set, and a test set is then composed of the remaining individuals (n_total_‐n_training set_). Here, we tested two proportions of individuals used in the training set (70% and 90%). Then, the assignment of the test set individuals was performed based on the information provided by the individuals in the training set. Since the actual origin of the used individuals is known, this analysis allows us to determine, at each iteration, if the information provided by the training set is able to correctly assign the test set individuals. Ultimately, this method provides an evaluation of the power of the dataset to properly assign individuals from the system under study to their population.

We expected an average assignment success per river of 90% or more, which is generally considered to be satisfying for fisheries management applications (e.g., Beacham et al., 2012, 2020; Seeb & Crane, 1999; Seeb et al., 2000). However, because the large number of low‐MAF markers introduced noise in the analysis, the dataset did not meet this threshold for samples from the GUE and DEL rivers (see Section 3, Figure 8a). In order to improve assignment precision, we created 15 SNP panels by sub‐setting the full dataset with varying numbers of SNPs (500, 1000, 2000, 3000…14,000). The procedure to create each of these panels was as follows: for each population pair, we sorted the loci by decreasing order of allele frequency difference (AFD) and then created lists of the most informative loci (larger AFD) for each population pair such that the sums of the squared AFDs in all of the lists were the same. As a result, the panels contain more informative loci for less differentiated populations, although with smaller average AFDs for less differentiated populations. This approach allowed for improved assignment success for the less differentiated populations, especially for the GUE and DEL rivers. Using these 15 panels, we then performed assignment tests, as described earlier, again using the 248 individuals from the source populations. The retained panel, containing 5000 SNPs, was found to maximize assignment success in each population while minimizing the variability of this success.

This dataset was then used as a reference for assigning fish captured in the estuarine mixed‐stock fisheries to their original population. The analysis was performed using the Bayesian approach implemented in the R package *rubias* v0.3.3 (Moran & Anderson, 2019) with 2000 iterations. Subsequently, only individuals for which the probability of assignment to one river was at least 10 times greater than the probability of assignment to any other river were kept.

## 3 RESULTS

### 3.1 Genotyping by sequencing

The final dataset consisted of 528 individuals, 248 of which were captured directly in the 8 studied rivers (source populations; average of *n* = 31 ± 11.39 per river) and 280 in the Koksoak R. estuary (mixed stock; Table 1). These individuals were genotyped with 14061 high‐quality filtered SNPs.

**TABLE 1:** Name and 3‐letter code of sampled rivers, geographic coordinates of sampling sites, and associated sample size used in analysis. Note that since there are multiple sampling sites in BAL R., the coordinates shown are those of the most downstream site.

Sampling site	Code	Longitude	Latitude	Sample size	Marine	Estuarine	Landlocked
Source populations							
Feuilles	FEU	−72.981	57.914	37	‐	‐	‐
Mélèzes	MEL	−71.883	57.155	30	20	7	3
Du Gué	GUE	−71.507	56.908	30	7	23	0
Delay	DEL	−71.082	56.519	42	11	31	0
Caniapiscau	CAN^a^	−69.502	57.599	19	0	13	4
Baleine	BAL	−67.706	57.904	39	‐	‐	‐
George	GEO	−64.975	56.836	41	‐	‐	‐
Nastapoka	NST	−76.537	56.911	10	‐	‐	‐
Mixed stocks (Koksoak estuary)							
2020	KOK^b^	−68.245	58.314	152	37	104	0
2021				128	42	36	0

^a^ In CAN R., there were also 1 juvenile and 1 mixed individuals.

^b^ In Koksoak estuary mixed‐stock fisheries, there were 8 mixed and 3 NA individuals in 2020, and 26 mixed and 24 NA in 2021.

### 3.2 Geographic population structure

**FIGURE 2:**
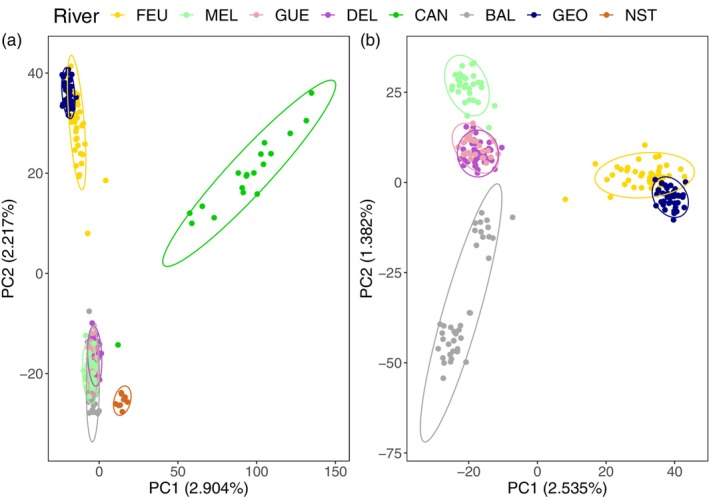
Results of principal component analysis showing PC scores for each individual along the first two principal axes for (a) all studied rivers and (b) the rivers that overlapped on the first two axes in (a) FEU, MEL, GUE, DEL, BAL, and GEO. Points representing individuals and 95% confidence ellipses are color‐coded by sampling location.

The PCA revealed the existence of genetic structure on the first two PC axes, which explained 2.904% and 2.217% of the variation in the data, respectively (Figure 2a). While the CAN and NST rivers clustered separately on these first two axes, the MEL, GUE, DEL, and BAL sites overlapped. The FEU and GEO rivers also showed some overlap, but to a lesser extent. In addition, CAN R. showed considerably more genetic variability than the other rivers on each of the two axes. Repeating this analysis, this time considering only the overlapping rivers (Figure 2b), showed that the MEL and BAL rivers clustered separately but that GUE and DEL, as well as FEU and GEO, were still overlapping. Finally, the results suggested the presence of genetic structure in the BAL R., with 2 observed clusters within this river (Figure 2b).

**FIGURE 3:**
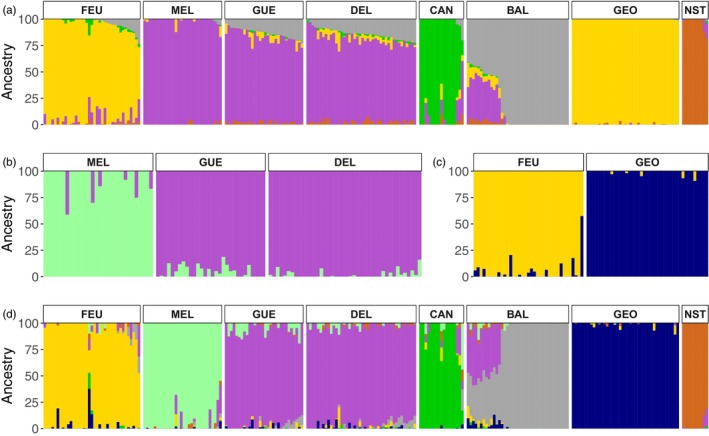
Admixture coefficient plots of the Bayesian clustering analysis using ADMIXTURE. The ancestry proportion (superposed) of each individual is represented by each vertical column. The results presented in (a) (*K* = 5) and (d) (*K* = 7) are from the same analysis run from *K* = 1 to 12, while only individuals from the MEL‐GUE‐DEL and FEU‐GEO groups were considered in (b) (*K* = 2) and (c) (*K* = 2), respectively.

Based on cross‐validation errors computed in ADMIXTURE, the best supported number of clusters was 5 when considering all sampled rivers (Figure S1a). At this K value, individuals from a given sampling location had a high coefficient of ancestry in the same cluster (Figure 3a). On average, individual membership in each river was above 85% (Figure 3a), with the exception of GUE (81.4%) and DEL (78.91%) rivers, as well as BAL (82.73%), where there were 12 first‐generation hybrids between BAL and GUE or DEL rivers (see *K* = 7, Figure 3d). Focusing on the MEL‐GUE‐DEL and FEU‐GEO clusters separately, two additional clusters were detected in each of these groups (Figure 3b,c), although the cross‐validation error for *K* = 1 was slightly lower in both cases (Figure S1b,c). This result was also observed with *K* = 7 using the whole dataset, which had a cross‐validation error value that was only slightly higher than *K* = 5. At *K* = 7, the average individual membership was generally greater than 90%, except for the CAN (86.03%) and BAL (79.94%) rivers, and we observed a similar pattern (compared to *K* = 5) of individuals from GUE R. (90.37%) and DEL R. (92.33%) belonging to the same cluster that is distinct from MEL R. Finally, except in BAL R. (Figure S1), we did not detect the presence of any clusters within each river when considered independently.

**FIGURE 4:**
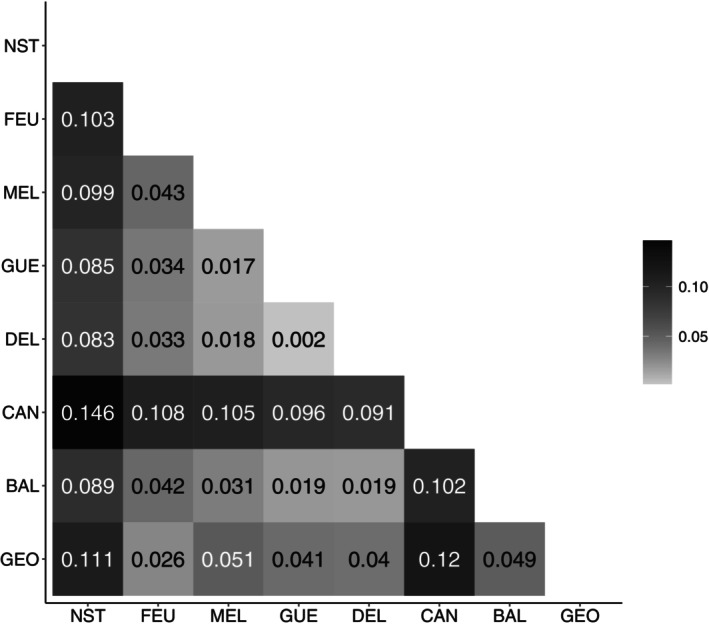
Heatmap of pairwise *F*_ST_ values; all values are significantly different from zero (*p* < 0.0001).

Average *F*_ST_ values between pairs of sampling sites were all significant (*p* < 0.001) and ranged from 0.002 to 0.12 in Ungava Bay's rivers and from 0.083 to 0.146 between NST R. and Ungava Bay's rivers (Figure 4). The lowest *F*_ST_ values between NST R. and Ungava Bay's rivers were with the GUE (0.085), DEL (0.083), and MEL (0.099) rivers, as well as with BAL R. (0.089).

Taken together, these results indicate the presence of 7 genetically distinct populations in Nunavik. Indeed, all the previously mentioned analyses showed that the FEU, MEL, CAN, BAL, GEO, and NST rivers are clearly differentiated from each other. Regarding the GUE and DEL rivers, while our results show that they are clearly differentiated from other Nunavik rivers, the structure between them is more ambiguous. Indeed, the PCA and ADMIXTURE results do not allow us to confidently assert that they are genetically distinct, but the *F*_ST_ value suggests weak differentiation. Thus, we consider that these rivers cannot be considered as clearly genetically distinct given our results.

### 3.3 Isolation‐by‐distance and gene flow

**FIGURE 5:**
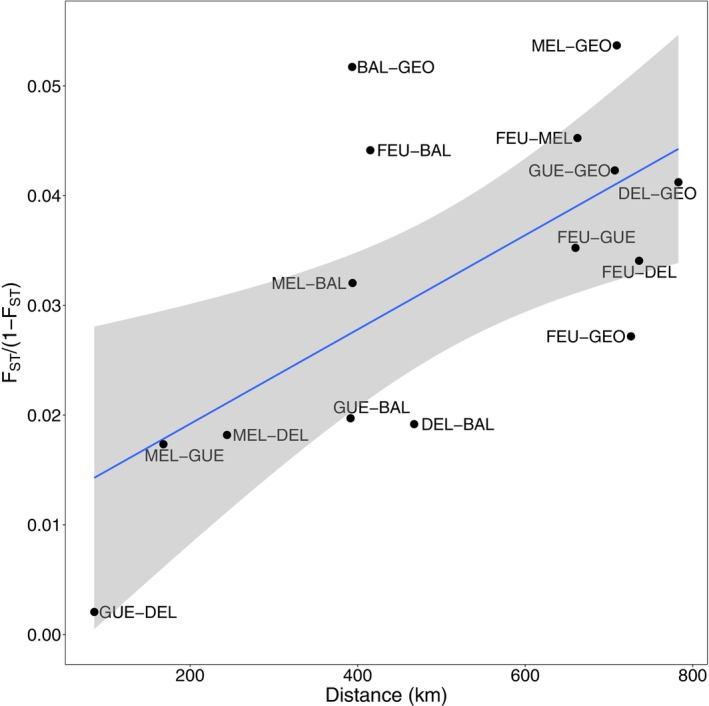
Isolation‐by‐distance represented by the relationship between linear *F*_ST_ values and geographic distance between sampling sites (considered as if a salmon was moving using the shortest distance between sites).

When all of Ungava Bay's rivers were considered in the isolation‐by‐distance model, the effect of distance on genetic differentiation was not significant (adjusted *R* ^2^ = −0.0253, *p* = 0.4852). In contrast, removing the CAN R. population improved the model fit, and the relationship was highly significant (adjusted *R* ^2^ = 0.3933, *p* = 0.0073). This latter model showed the lowest AICc value (Table 2), suggesting that IBD explained a large proportion of the genetic structure in Ungava Bay (Figure 5). Notably, the FEU‐BAL and BAL‐GEO comparisons had high *F*_ST_ values for a relatively small geographic distance. Conversely, the FEU‐GEO comparison showed a relatively low *F*_ST_ for a large geographic distance. Moreover, all three comparisons are outside the 95% confidence interval, suggesting that factors other than geographic distance may influence population genetic structure, particularly in the FEU and GEO rivers.

**TABLE 2:** Parameters associated with the isolation‐by‐distance (IBD) models, including the degrees of freedom (DF), Akaike information criterion value with correction for small sample sizes (AICc), adjusted *R* ^2^, and *p* values. “All Ungava R.” includes FEU, MEL, GUE, DEL, CAN, BAL, and GEO rivers, while “All Ungava R. but CAN” includes the same rivers but excludes CAN R. *F*_ST_~0 are null models.

Rivers considered	Model	df	AICc	*R* ^2^	*p* value
All Ungava R. but CAN	*F*_ST_~Dist	13	−85.47205	0.3933	0.0073
All Ungava R.	*F*_ST_~Dist	19	−68.57069	−0.0253	0.4852
All Ungava R. but CAN	*F*_ST_~0	15	−50.47494	NA	NA
All Ungava R.	*F*_ST_~0	21	−50.47494	NA	NA

**FIGURE 6:**
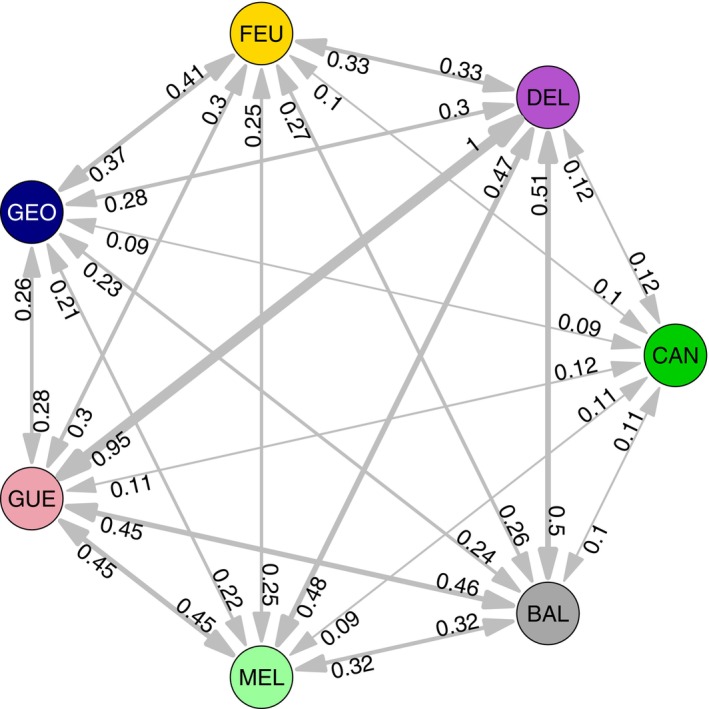
Directional relative gene flow between pairs of rivers within Ungava Bay. NST R. was not included since it is considered completely isolated, probably since the last glaciation. Arrow widths represent the intensity of the relative gene flows (values on arrows).

Finally, the evaluation of relative migration showed that gene flow is generally higher between rivers within the Koksoak system (MEL, GUE, and DEL rivers) as well as between Koksoak rivers and BAL R. In particular, within the Koksoak system, migration is especially high between the GUE and DEL rivers (Figure 6). In contrast, gene flow between CAN R. and all other sites is relatively low, despite also being within the Koksoak system. Furthermore, there is more exchange between FEU and GEO rivers than between FEU or GEO rivers and any other river.

### 3.4 Genetic diversity within sampling sites

In Ungava Bay's rivers, He and Ho varied slightly, ranging from 0.216 to 0.217 and from 0.191 to 0.214, respectively (Table 3). Except for CAN R., which had a significantly lower Ho than the other rivers (Tukey test, *p* < 0.017), there were no significant differences (*p* > 0.05) in He or Ho between any of Ungava Bay's rivers. The NST R., which flows into Hudson Bay rather than Ungava Bay, showed significantly higher genetic diversity than the other rivers (Tukey test, *p* < 0.0001), but a considerably higher Ho (0.29 ± 0.48) than He (0.215 ± 0.0009). CAN R. showed the lowest effective population size (*N*_e_) (49) by far, while that of the other rivers ranged from 445 (BAL R.) to 11,496 (GUE R.; Table 3) and none of the confidence intervals overlapped. The sample size was too small (*n* = 10) to calculate N_e_ in the NST R. (Do et al., 2014).

**TABLE 3:** Summary of descriptive statistics: observed heterozygosity (Ho), expected heterozygosity (He), *F*_IS_, and effective population size (*N*_e_); SD refers to the standard deviation and CI refers to the confidence interval.

Code	Ho (SD)	He (SD)	*F*_IS_ (SD)	N_e_ (CI)
FEU	0.207 (0.008)	0.217 (0.0003)	0.044 (0.037)	1197 (1142.3–1260.8)
MEL	0.206 (0.014)	0.217 (0.0005)	0.05 (0.064)	612 (593.9–632.1)
GUE	0.211 (0.008)	0.216 (0.0003)	0.025 (0.04)	11,496 (7372.7–26054.6)
DEL	0.214 (0.007)	0.216 (0.0004)	0.013 (0.032)	4975 (4223.4–6050.3)
CAN	0.191 (0.028)	0.217 (0.0002)	0.12 (0.131)	49 (48.7–49.2)
BAL	0.214 (0.01)	0.217 (0.0006)	0.012 (0.045)	445 (437.3–452.7)
GEO	0.205 (0.005)	0.217 (0.0003)	0.052 (0.024)	1075 (1031.9–1120.7)
NST	0.290 (0.048)	0.215 (0.0009)	−0.349 (0.226)	NA

### 3.5 Genetic basis of life‐history tactics

The analysis of molecular variance (AMOVA; Table 4) as well as a PCA (Figure S3) showed that structure between rivers, largely driven by differentiation between MEL versus GUE and DEL rivers, explained a significant component (*p* = 0.001) of the variation (>1.267%), while structure between life‐history tactics did not (*p* = 0.633). At the second hierarchical level, differentiation between rivers within each life‐history group explained more variation (1.346%) than differentiation between life‐history groups within rivers (0.103%). In order to limit the noise associated with inter‐river structure, MEL R. was subsequently removed from the dataset. Both the GUE and DEL rivers were retained due to the high genetic similarity between these two sites, bringing the sample size to 272, including individuals caught within the estuary and assigned to these 2 rivers.

**TABLE 4:** Analysis of molecular variance (AMOVA) results of population and life‐history tactic structure, with river and life‐history tactic as first and second hierarchical levels in the first model, respectively, and vice versa in the second model. The analyses were run with individuals caught in the MEL, GUE, and DEL rivers, as well as those caught within the estuary and assigned to these three rivers. Gen refers to a Genind class object, or, in other words, to the genetic data matrix (14,061 SNPs genotyped in 304 individuals).

Model	Source of variation	df	Sum of squares	Percentage of variation	*p* value
Gen~River/Life‐history tactic	Between rivers	2	6126.104	1.267	0.001
	Between life‐history tactics, within the river	3	4494.629	0.103	0.013
	Within life‐history tactics	298	426360.279	98.630	0.001
Gen~Life‐history tactic/River	Between life‐history tactics	1	1645.272	−0.491	0.633
	Between rivers, within life‐history tactics	4	8975.461	1.346	0.001
	Within rivers	298	426360.279	99.146	0.001

**FIGURE 7:**
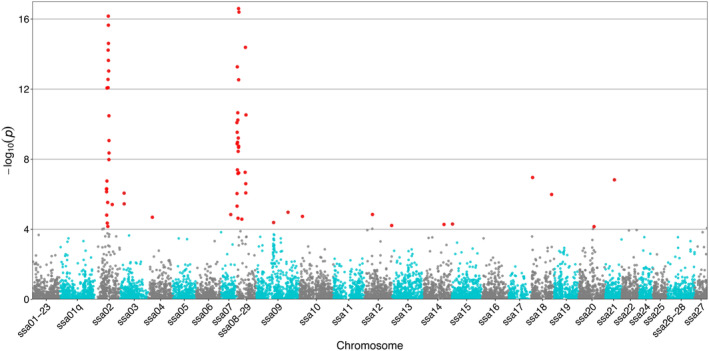
Manhattan plot showing the distribution of candidate outlier SNPs (red dots) associated with estuarine and marine life‐history tactics in GUE and DEL rivers along the genome, using a *q* value of 0.01 as the alpha threshold from PCadapt. Turquoise and dark gray colors alternate between chromosomes for clarity.

Using these samples, PCadapt identified a total of 63 SNPs significantly (*q* value<0.01) associated with life‐history tactics, including 22 on chromosome ssa02 and 26 on the previously documented fusion (Wellband et al., 2019) between chromosomes ssa08 and ssa29 (ssa08‐29; Figure 7). Using only these SNPs increased the average *F*_ST_ between life‐history tactics by 30 times, raising it from 0.00028 (using all 14,061 SNPs) to 0.01022 (using the 63 SNPs associated with life‐history tactics). While this suggests that these SNPs are associated with life‐history tactics, the value is still weak, meaning that they are far from being fixed. That being said, those SNPs allowed the identification of genes localized within 20 kb from outlier SNPs identified by PCadapt (Table S2), of which 9 GO biological processes were significantly enriched (*p* < 0.05) on chromosomes ssa02, ssa08‐29, and ssa21 (Table 5). Moreover, genes related to growth (response to nutrient levels, bone and cartilage development, ossification, organ morphogenesis, and larval heart development), temperature response, and brain development (pituitary gland development, modulation of synaptic transmission, neurogenesis, brain morphogenesis, dendrite morphogenesis, regulation of synaptic structural plasticity, and regulation of synaptic plasticity) were located within 1 kb of outlier SNPs on chromosomes ssa02 and ssa08‐29.

**TABLE 5:** Gene ontology enrichment for genes located within a region of 20 kb around outlier SNPs identified by PCadapt.

GO ID	Term	*p* value	Chromosome
GO:0030036	Actin cytoskeleton organization	0.032	ssa08‐29
GO:0061061	Muscle structure development	0.032	ssa08‐29
GO:0007507	Heart development	0.033	ssa08‐29
GO:0051209	Release of sequestered calcium ion into cytosol	0.044	ssa02
GO:0009267	Cellular response to starvation	0.046	ssa21
GO:0030150	Protein import into mitochondrial matrix	0.046	ssa21
GO:1902957	Negative regulation of mitochondrial electron transport	0.046	ssa21
GO:0031333	Negative regulation of protein‐containing complex assembly	0.046	ssa21
GO:0019216	Regulation of lipid metabolic process	0.047	ssa21

### 3.6 Genetic stock identification and contribution of source populations in mixed‐stock fisheries

**FIGURE 8:**
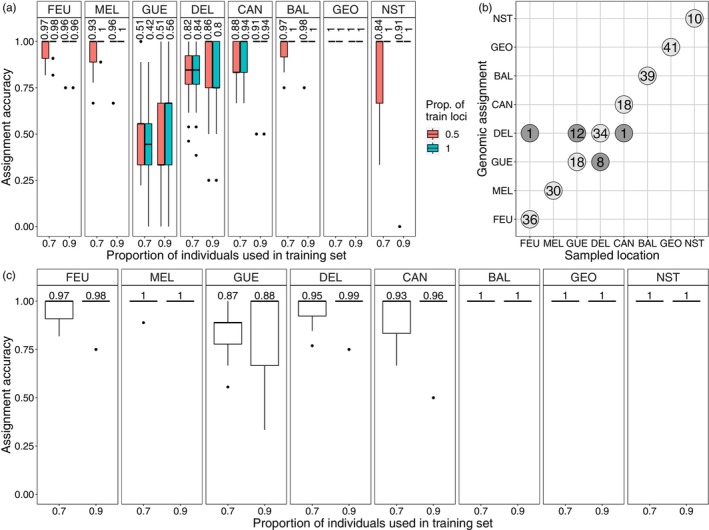
(a) Boxplot showing the assignment accuracy of salmon from the 8 source populations, as estimated by Monte–Carlo cross‐validation using all 14,061 SNPs, including two proportions of training individuals (70% and 90%) and training loci (50% and 100%) with 100 iterations. The values above the boxes indicate the average assignment success. (b) Number of individuals correctly assigned (light gray circles on diagonal) and incorrectly assigned (dark gray above and below diagonal) to their population of origin. Sample collection (*x*) corresponds to the river of origin, and Genomic assignment (*y*) corresponds to the assigned river. (c) Boxplot showing the assignment accuracy of salmon from the 8 source populations estimated by Monte–Carlo cross‐validation, with a subsample of 5000 SNPs selected in order to maximize the allele frequency difference (AFD) between pairs of populations, including two proportions of training individuals (70% and 90%), with 100 iterations. The values above the boxes are the average assignment success.

When the GUE and DEL rivers were treated as a single population using all 14,061 SNPs, assignment success was 100% (data not shown), but when these rivers were considered as separate, assignment success was lower (GUE≤56%, DEL≤86%), and most assignment errors involved these two rivers (Figure 8a,b). However, using a subsample of 5000 SNPs enabled us to achieve an assignment success of 88% for GUE and 99% for DEL when they were treated separately (Figure 8c)—with 100% success when considered as a single population (data not shown). Although the 90% threshold was not met for the GUE river, we chose to consider them as two populations for the assignment of individuals caught within the estuary to describe the contribution of each river with as much precision as possible. This will probably lead to some misassignments, but these are likely to occur mainly between GUE and DEL. Finally, when using the 5000 SNPs dataset and considering GUE and DEL as two distinct populations, 3 individuals caught in the estuary and assigned to their river of origin were excluded as they did not meet our criterion of having a 10‐fold higher assignment probability compared to other rivers.

**FIGURE 9:**
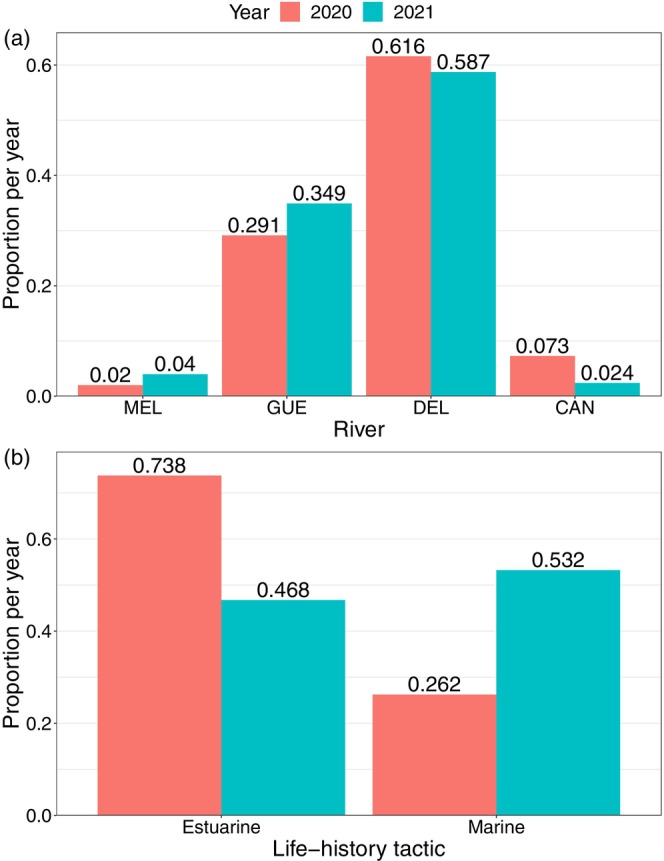
(a) Assignment results of the 280 individuals (of which 3 were removed because they did not meet assignment criteria) caught in the estuarine mixed stocks fisheries and contribution of rivers to the subsistence fisheries, for the two sampling years, based on the dataset used in Figure 8c. (b) Contribution of estuarine and marine life‐history tactics, identified by scale reading (*n* = 218), in estuarine fisheries for both sampling years. The values above the bars are the proportion of individuals associated with that category (river or life‐history tactic) for that year.

During both sampling years, the GUE and DEL rivers together contributed to the majority of the salmon caught in the estuary, accounting for 29.1% and 61.6% of the 2020 fish and 34.9% and 58.7% of the 2021 captures, respectively (Figure 9a; Table S3). The contribution of the DEL R. in the estuarine fisheries was almost double that of the GUE R. The MEL and CAN rivers, respectively, contributed 2% and 7.3% of the 2020 captures and 4% and 2.4% in 2021. No significant inter‐annual variation in the contribution of populations to the mixed fisheries was observed (chi‐square test, *p* = 0.1705). Conversely, significant inter‐annual variation (chi‐square test, *p* < 0.00001) was observed between marine and estuarine life‐history tactics (Figure 9b). While estuarine salmon contributed 73.8% to the fisheries in 2020, they contributed only 46.8% in 2021 (Table S2). For marine salmon, the contribution was 26.2% and 53.2% in 2020 and 2021, respectively.

## 4 DISCUSSION

This study represents the first fine‐scale population genomics and life‐history tactics study of the northernmost Atlantic salmon in North America. It also represents the first investigation of the contribution of each population and of the estuarine and marine migratory life‐history tactics in the Koksoak subsistence fisheries that are of great importance for the indigenous people of the region. Our findings revealed that Nunavik harbors 7 genetically distinct populations out of the 8 rivers considered and that the extent of differentiation between rivers is primarily driven by isolation‐by‐distance (IBD) and patterns of gene flow between rivers. Moreover, the absence of a neutral structure between estuarine and marine salmon present in the Koksoak R. system, coupled with the fact that two genomic regions are putatively associated with migration tactics, suggests a genetic basis for tactic determination within a population. Finally, genetic stock identification (GSI) analysis showed that the vast majority of salmon caught in mixed‐stock fisheries within the Koksoak R. estuary originate from the same two rivers and that there is no inter‐annual variation in the origin of these salmon. However, our results show that both estuarine and marine life‐history tactics contribute significantly to these fisheries and that there appears to be inter‐annual variation in this contribution.

### 4.1 Population structure and gene flow

Our results revealed a hierarchical structure in which each of the 5 main studied rivers (FEU, Koksoak, BAL, GEO, and NST) is genetically distinct. Similarly, the 4 rivers sampled within the Koksoak system (MEL, GUE, DEL, and CAN) constitute 3 distinct populations (MEL, GUE‐DEL, and CAN). As previously mentioned, our results do not strongly support the presence of genetic structure between the GUE and DEL rivers and therefore do not allow us to assert their evolutionary independence. However, our ability to assign individuals from these sampling sites with a success rate of at least 87% suggests that these two rivers do not constitute a completely panmictic population. Therefore, they may experience demographic independence, which implies that local contemporary recruitment may have a stronger impact on the population size and genetic diversity of each river than the migration rate between those. It seems most likely that our SNPs dataset expresses evolutionary processes rather than contemporary processes and may not be suitable to detect such a current demographic phenomenon. Demographic inferences based on coalescence should be conducted to estimate current demographic parameters between these two rivers before we can conclude on their demographic independence (e.g. Dorant et al., 2022; Ferchaud et al., 2022). We therefore recommend that, in a management context, it is best to proceed with caution and consider these rivers as two populations at least until more data (e.g., based on coalescence analysis) is available on their demographic independence.

Nevertheless, the hierarchical genetic structure observed in Nunavik salmon rivers is consistent with our expectations given the species' strong homing behavior, which reduces gene flow from one river to another (Keefer & Caudill, 2014; Quinn, 2018) and thus increases divergence. Many studies have observed a hierarchical pattern of population structure in Atlantic salmon (Bourret et al., 2013; Dionne et al., 2008, 2009; Moore et al., 2014; Perrier et al., 2011). Also, a pattern of population structure where the sub‐watersheds within a system are less divergent from each other than they are from neighboring systems has also been reported in several large systems (Aykanat et al., 2015; Dionne et al., 2009; Primmer et al., 2006). An important exception is the Miramichi R., which is the largest salmon river in North America and where no genetic structure has been observed (Dionne et al., 2009; Wellband et al., 2019).

Our results showed that IBD largely explains the genetic structure observed in Ungava Bay and are consistent with other studies on Atlantic salmon demonstrating IBD at varying scales, range‐wide (King et al., 2001), between regions (Dillane et al., 2007; McConnell et al., 1997; Perrier et al., 2011; Säisä et al., 2005; Tonteri et al., 2009), and within the same system (Primmer et al., 2006). In Ungava Bay, this relationship between geographic distance and genetic differentiation was only observed when the CAN R. was removed from the analysis. Although the CAN R. is also located within the Koksoak system, it is the most divergent population in Ungava Bay. The most likely explanation is that our sampling represents a stretch of about 28 km (corresponding to about 3% of the river's entire length) and was located downstream of the Limestone Falls, which is impassable for salmon migrating upstream. However, the presence of a landlocked salmon population upstream of the falls is known. Since migration into this population has probably been impossible since the last Ice Age (5500–6000 years BP; Dalton et al., 2020), it is likely strongly affected by genetic drift. As a result, it is expected to have low genetic diversity and to be highly differentiated from other populations in the system (e.g., Sandlund et al., 2014; Whiteley et al., 2010). Low *N*_e_, as well as low Ho, suggests that the strong differentiation between the CAN R. site and the others could be caused by unidirectional gene flow from this isolated population.

Although the results showed that FEU and GEO rivers are genetically distinct, the extent of differentiation between these two rivers was relatively weak and they were more genetically similar to each other than either of them was to any other population in Ungava Bay. Although the possibility of catching more migrants between the FEU and GEO rivers by chance cannot be ruled out, our results suggest that migration is greater between these two rivers than between either of them and any other river in Ungava Bay, which is likely to maintain genetic differences between the FEU‐GEO complex and the Koksoak‐BAL complex (MEL, GUE, DEL, and BAL). Several hypotheses can explain this reduced exchange between these two river complexes. First, the presence of estuarine salmon in the Koksoak R. might limit migration from the Koksoak to other rivers since these salmon do not leave the river, thus maintaining divergence between these two complexes. However, the magnitude of this effect is not clear for FEU, BAL, and GEO rivers, where the proportion or occurrence of estuarine salmon is unknown. In any case, the presence of Arctic char in FEU and GEO rivers (Dallaire et al., 2021; Dubos et al., 2022) (unknown in BAL R.), while absent from the Koksoak (Robitaille et al., 1986), may limit the occurrence of estuarine salmon. Second, despite the limited availability of environmental data, the northerly location of the FEU R. and the presence of the Torngats Mountains in the GEO R. watershed result in more arctic habitats compared to Koksoak and BAL rivers (MFFP, 2018). In addition, the southern source of the CAN, which represents over 50% of the Koksoak watershed, and BAL rivers is likely to result in higher overall water temperatures in the Koksoak and BAL rivers. The GEO R. has its source in the south, but the presence of the Torngats Mountains is likely to cool the water. River chemistry, which can be influenced by the vegetation in the watershed (Thomas, 1997), and temperature are known to be major components in river identification during the return migration of adult salmon (Keefer & Caudill, 2014). Thus, the difference in physicochemistry between the two river complexes could increase patterns of migration within them and minimize them between them.

We provide the first genetic study of the BAL R., which is genetically different from the other Ungava Bay rivers. Although it is genetically similar to the MEL, GUE, and DEL rivers, we still consider BAL R. to be a distinct population since PCA and ADMIXTURE are able to differentiate it. Also, *F*_ST_ values between BAL and MEL/GUE/DEL are similar to those between MEL and GUE/DEL. Finally, whether we use the whole set of SNPs or a subsample of SNPs, assignment success is at least 97% for BAL individuals. In addition, the finding that BAL R. is more similar to MEL, GUE, and DEL (compared with pairwise *F*_ST_ with other rivers), combined with the fact that migration is strong between the BAL R. and these three rivers, is consistent with IBD. Furthermore, as mentioned above, the BAL and the Koksoak rivers have similar environments in terms of forest composition and temperature. While the identification of 12 first‐generation hybrids between BAL R. and GUE or DEL rivers in BAL could suggest that there is substantial migration between these rivers, it could also result from the particularities of our sampling. In BAL R., we sampled fry and parr stages, which move very little, and this could have led to an overrepresentation of highly related individuals, like full‐sibs or half‐sibs, that may have reduced the measured differentiation.

Finally, we provide the first genetic study of salmon from the NST R., the only known river in Hudson Bay harboring a self‐sustaining population of landlocked salmon, above the impassable 35‐m‐high Nastapoka Fall (April et al., 2023; Le Jeune & Legendre, 1968). According to Morin (1991), salmon may have colonized the NST R. during the last deglaciation period (6000–5500 BP; Dalton et al., 2020) via the Lac des Loups Marins from the MEL R. Our results are in line with this hypothesis. Indeed, based on *F*_ST_ values, the MEL, GUE, and DEL rivers are the least divergent from the NST R., compared with the other studied rivers. However, the fact that the *F*_ST_ between the NST and the MEL (0.099) rivers is higher than the *F*_ST_ values between the NST and the GUE (0.085) and DEL (0.083) rivers suggests that colonization may also have occurred via one of the latter two rivers.

### 4.2 Evidence for a genetic basis of migratory life‐history tactics

Our results showed that both migratory life‐history tactics (marine and estuarine) are present in each of the MEL, GUE, and DEL rivers. The extremely weak *F*_ST_ values between the two life‐history tactics when all SNPs were used, in addition to the absence of structure in PCA and ADMIXTURE, suggest random mating between migratory life‐history tactics within each tributary. The presence of two or more life‐history tactics in an apparently panmictic population has previously been observed in Atlantic salmon (Adams et al., 2016) and in other salmonid species, including rainbow trout (*Oncorhynchus mykiss*; McPhee et al., 2007; Olsen et al., 2006), brown trout (*Salmo trutta*; Charles et al., 2005; Hindar et al., 1991), bull trout (*Salvelinis confluentus*; Homel et al., 2008), and cutthroat trout (*Oncorhynchus clarki*; Johnson et al., 2010; Whiteley et al., 2010). In addition, the identification of significant outlier SNPs associated with these marine versus estuarine life‐history tactics in salmon indicates the presence of a putative genetic determinism of migratory life‐history tactics. Considering the relatively small number of SNPs, results related to identified functional traits, whether significant or not, should be interpreted with caution, and analyses based on whole‐genome sequencing would be required to find all potential genetic markers of interest and the genes they potentially affect. Nonetheless, we identified several genes located close to the outlier SNPs, and these are linked to various biological functions that may have an impact on the timing of smoltification, which both estuarine and marine salmon have to undergo in order to reach the estuary or sea. This includes, for instance: growth, response to temperature, brain development, and light detection. Furthermore, this is consistent with the findings of Wellband et al. (2019), who identified genes on the chromosomal fusion between chromosomes ssa08 and ssa29 whose function is related to neuron development, phototransduction, cell adhesion (thus possibly linked to growth), and aging using a dataset roughly 4× larger than the one used here (52,537 SNPs). To our knowledge, there is currently no available information regarding the possible functional role of SNPs identified on chromosome ssa02 in Atlantic salmon in the genomic region where we observed a strong association with migratory life‐history tactics. Further analysis, notably using a quantitative genetic approach, would be required to accurately measure the genetic contribution to life‐history tactics.

Among the genes that we identified in genomic regions putatively associated with life‐history, some were found to be linked to growth rate. Given that fish length is mainly used as a threshold trait when deciding whether to migrate or to suspend migration (Dodson et al., 2013)—since it has a determining effect on age at smoltification (Jonsson & Jonsson, 2011)—the identified traits might have an effect on reaching the threshold for migration initiation (or smolting) within a season. That may then impact when smolts enter the estuary and, therefore, when they have the possibility to enter the sea, ultimately having an influence on the determination of the migratory tactic. Typically, individuals with a higher growth rate migrate at a younger age, while those with a lower growth rate delay their migration to a later season. However, Jensen et al. (2012) showed that size can also impact the timing of migration within a season: during the first half of the smoltification period, they observed an increase in individual size, followed by a decrease in size during the second part of the season. They suggest that the fish that underwent smoltification in the first half of the season had higher metabolic requirements (Rikardsen & Elliott, 2000), while the remaining fish had to wait a few weeks to complete their growth and migrate at the end of the season. Together, these findings suggest an indirect genetic control of migration in which the adopted migratory tactic is affected by an individual's physiology, which is in turn influenced by its genotype (Kelson et al., 2020). Our results are also consistent with Power's (Power, 1981) hypothesis that life‐history tactic determination in Ungava Bay populations depends on when smolts are able to enter the sea. Indeed, since the time window in which temperature allows smolts to enter the sea in Ungava Bay is very short, late migrants are likely to get stuck in the estuary and have no choice but to adopt an estuarine tactic. Additional studies could investigate the potential role of water temperature, spatially variable selection, and genotype‐dependent habitat choices in this system (Campbell et al., 2022; Pavey et al., 2015).

### 4.3 Contribution of populations and life‐history tactics in mixed‐stock subsistence fisheries

Our results pertaining to the contribution of Ungava Bay salmon populations to subsistence fisheries in the Koksoak R. estuary indicate that the majority (>90% in both years) of salmon caught in the estuary originate from the GUE (>29.1%) and DEL (>58.7%) rivers. As previously mentioned, the exact contribution of both GUE and DEL rivers taken individually is possibly slightly biased since assignment success is weaker for source individuals (especially for GUE: 88%). However, if there are misassigned individuals originating from one of these two rivers, we are confident that they come from either GUE or DEL R. In other words, an individual wrongly assigned to GUE R. likely comes from DEL R., and vice versa for an individual assigned to DEL R. The greater contribution of the GUE and DEL rivers to the estuary could be driven by the habitat in these rivers, which is considerably more favorable to salmon reproduction compared to the portion of MEL and CAN that is accessible to anadromous salmon (MFFP, 2015). Furthermore, the large effective population sizes from the latter two rivers compared to the MEL and CAN populations indicate that the effect of genetic drift is less important there (Waples, 2022), suggesting larger population sizes and explaining their greater contribution to estuarine fisheries. To explain the great difference in contribution between GUE/DEL and MEL/CAN, we considered the possibility that fish from MEL and CAN migrate at a different time or occupy a different portion of the estuary than those from GUE and DEL. However, although the migration distance is shorter in CAN R. and in the downstream portion of MEL R. compared to the GUE or DEL rivers, we have no reason to believe that salmon from the MEL or CAN rivers migrate at a different time than those from the GUE and DEL rivers. Moreover, even if they did migrate at different times, we have no reason to believe that they occupy different parts of the estuary. Thus, in terms of the location of fisheries in the estuary, there is currently no evidence to suggest that individuals from one river might be fished disproportionately to those from other rivers.

Secondly, our results show that 100% of the individuals caught in the estuary come from the Koksoak R. system. Considering the species' strong homing behavior, we expected the majority of estuarine catches to come from the Koksoak watershed. However, it would not have been surprising to observe at least a few migrants from neighboring rivers. For example, salmon from the United States have been captured in salmon river estuaries in Labrador, Canada (Bradbury et al., 2015) and along the Greenland coast (Gauthier‐Ouellet et al., 2009), and individuals from 3 different rivers from the St. Lawrence R. North Shore region (Québec, Canada) have been captured in Vieux‐Fort R. mouth (Colombani et al., 1998). This is nearly 2000, 3000, and 500 km from their river of origin, compared to less than 200 km between Ungava Bay's rivers. This emphasizes that migration between Ungava Bay's rivers is low and that salmon from other rivers are unlikely to use the Koksoak estuary as a feeding area in the same way that estuarine salmon from the Koksoak R. do in this estuary. Finally, we did not observe inter‐annual variation in the origin of fish caught in the estuary. Admittedly, only 2 years of sampling were available for this analysis, and it is conceivable that the contribution of these rivers is more variable than we observed. Yet, the large contribution of the GUE and DEL rivers, compared with the MEL and CAN rivers, is clear. It is also evident that the contribution of the other rivers (FEU, BAL, and GEO) is low, although a negligible contribution from these systems is quite realistic.

We observed significant inter‐annual variation in the contribution of both life‐history tactics to estuarine fisheries. But again, the study was conducted over only 2 years, and the 2020 sampling period ran from June 25 to October 1, whereas the 2021 sampling period ran from August 14 to September 14. Given that marine salmon do not enter the river until late July or even early August (Robitaille et al., 1982, 1984, 1986), this is a potential bias in the proportions of each life‐history tactic. That being said, it remains that the majority of the 2020 catches (>70%) were made during the same period as 2021. Furthermore, Robitaille et al. (1982) reported that in estuarine catches made by Inuit from mid‐July to September, 30% were estuarine salmon versus 70% marine salmon, which is significantly different from our catches in both 2020 (*p* < 0.0001) and 2021 (*p* = 0.0048). Robitaille et al., (Robitaille et al., 1982) also found significant inter‐annual variation in the DEL R. from 1979 to 1981. This leads us to believe that inter‐annual variation in life‐history tactics is indeed a feature of these fisheries.

Nevertheless, it is clear that the contribution of both migratory life‐history tactics to estuarine subsistence fisheries is important. Inuit from Kuujjuaq mainly target marine salmon, which are generally larger (Robitaille et al., 1986), and this is why estuarine fisheries mainly take place in August. That being said, even in the month of August, although the main focus is on marine salmon, estuarine salmon still represents a substantial proportion of the harvest.

### 4.4 Implications for conservation and management

In a context where salmon represents an important source of harvested food as well as economic and cultural resources for Ungava Bay's Inuit communities, especially in Kuujjuaq, the information provided in this study will contribute to informed management decisions that will be made in a concerted manner between the indigenous communities and the government. In concrete terms, given the presence of one population per river (whether they are evolutionarily independent or possibly demographically independent), management should be carried out river by river (or tributary by tributary), as is typically done in Quebec (MFFP, 2016). Moreover, we now know that salmon from the GUE and DEL rivers account for almost all catches in the Koksoak system. Indeed, the majority of outfitters in this system are located there, and these rivers produce the vast majority of the salmon caught by the Inuit of Kuujjuaq. We therefore consider that these populations should be management priorities. This is all the more relevant given that no fish from the FEU, BAL, and GEO rivers have been found in estuarine fisheries. By keeping the GUE and DEL rivers a priority and by considering them as two populations despite ambiguity from a genetic point of view, the sustainability of the estuarine subsistence fisheries would be maximized, and so would food security.

This study also indicates that both estuarine and marine salmon are present in almost all rivers of the Koksoak (although with less certainty in the CAN R.). Furthermore, our results support the hypothesis of an indirect genetic basis for these migratory tactics, implying the possibility of fishery‐induced selection (Thériault et al., 2008) since the fishery in the Koksoak R. estuary may not apply the same pressures equally to these two tactics. Indeed, the Inuits of Kuujjuaq are mainly interested in marine salmon, which are generally larger than their estuarine counterparts (Robitaille et al., 1982, 1984, 1986). Additionally, Robitaille et al. (1982) showed that marine salmon tend to feed in rivers, which leads us to hypothesize that they may be more inclined to bite in recreational fisheries compared to estuarine salmon. Since the presence of these tactics is likely to have a substantial impact on the survival of the species in this harsh environment (Robitaille et al., 1986), we consider that management actions should aim to protect this intra‐population diversity by considering both the estuarine and marine contingents. Studies looking at the evolution of these migratory tactics in the context of fishing pressure and even in the context of climate change are therefore required to promote the conservation of this intra‐specific diversity and ultimately foster the sustainability of the fisheries and food security of native communities.

## CONFLICT OF INTEREST STATEMENT

We declare that there are no conflicts of interest.

## Supporting information


Figures S1–S3.

Tables S1–S3.

Data S1.

## Data Availability

Individual read raw sequences will be available at the Sequence Read Archive (SRA) (Project Accession Nos.: PRJNA1039799) upon acceptance.
