# Supplementary material for: Population genomics, life‐history tactics, and mixed‐stock subsistence fisheries in the northernmost American Atlantic salmon populations
Source: Evol Appl. 2024 Feb 22;17(2):e13654. doi: 10.1111/eva.13654 (PMC10883791; doi:10.1111/eva.13654)

Supplementary Figure 1 Cross-validation (CV) error for an ADMIXTURE clustering analysis according to the number of considered clusters (K) in A) all studied rivers (K=1-12), B) only Mélèzes, Du Gué and Delay rivers (K=1-5), and C) only in Feuilles and George rivers (K=1-5).

Supplementary Figure 2 Genetic structure within the Baleine R. (BAL), associated with sampling sites and represented by A) a PCA showing PC scores for each individual (n=39) along the first two axes (4.733% and 3.339% of total variance explained, respectively), and C) a barplot of admixture coefficients from the Bayesian cluster analysis from ADMIXTURE. B) A map depicting the sampling sites location along the river.

Supplementary Figure 3 Results of principal component analysis showing PC scores for each individual along the first two principal axes (0.942% and 0.679% of the total variance explained, respectively) using estuarine and marine life-history strategies from Mélèzes, Du Gué and Delay rivers, which are represented by colors and shape, respectively.


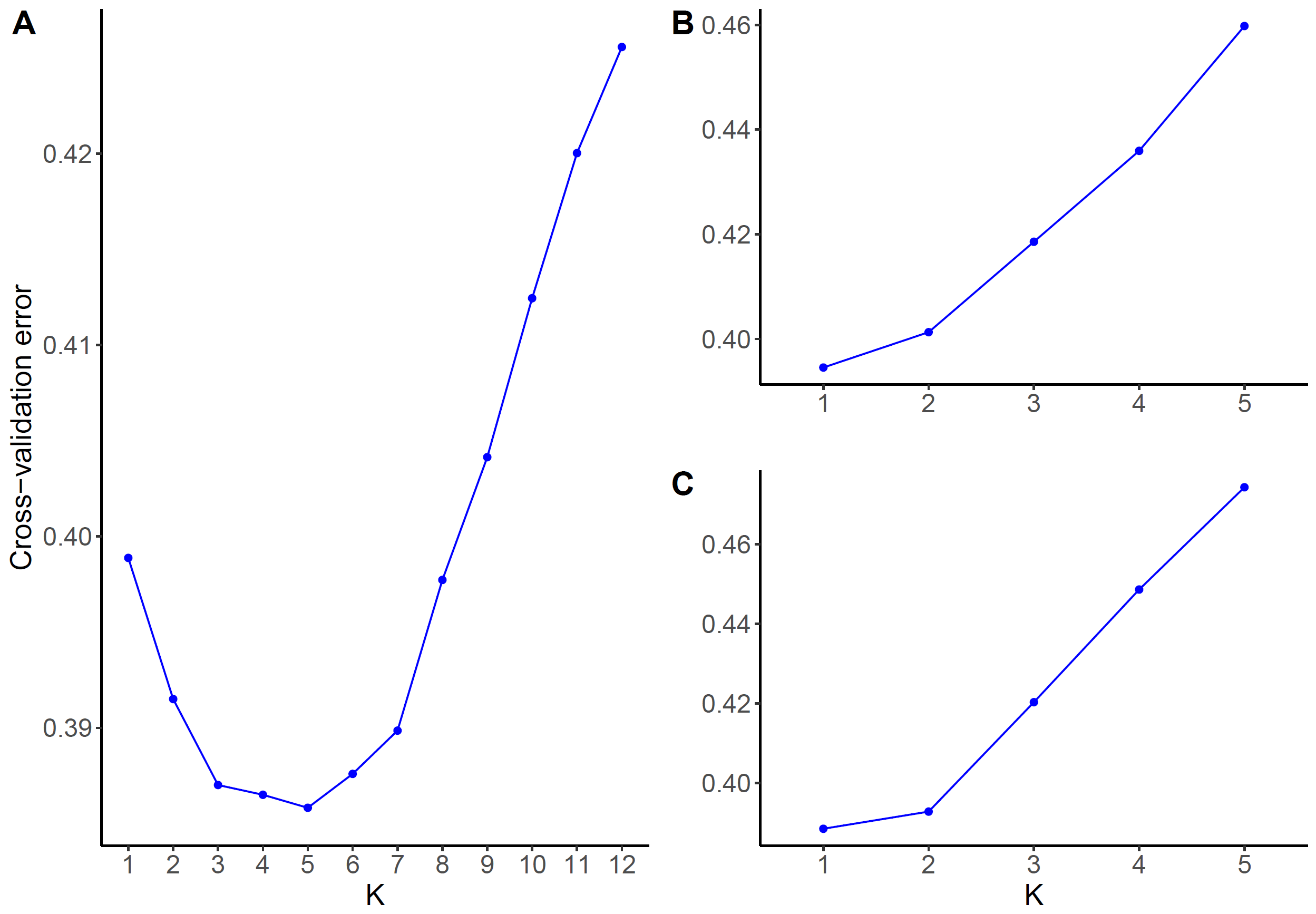


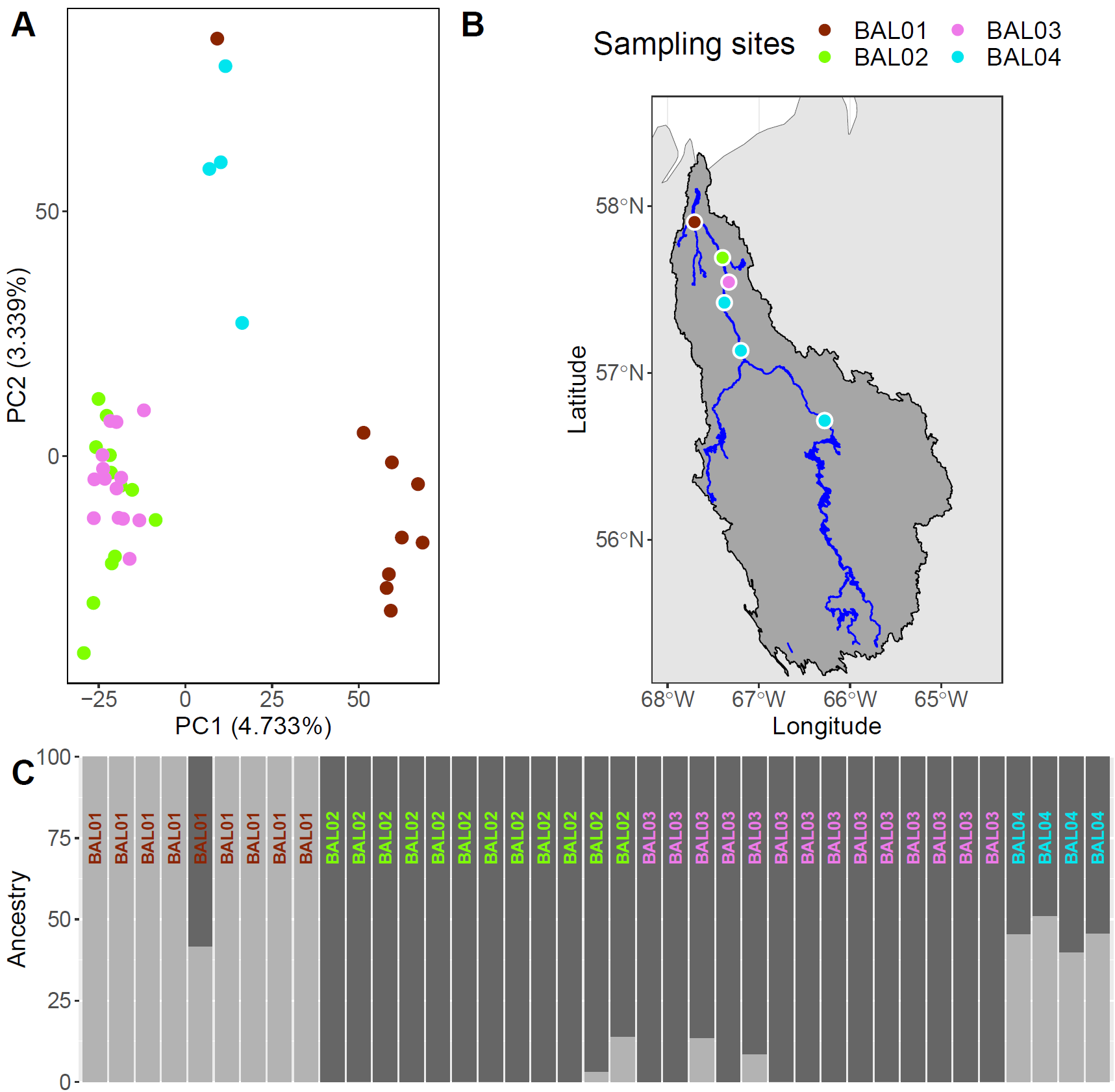


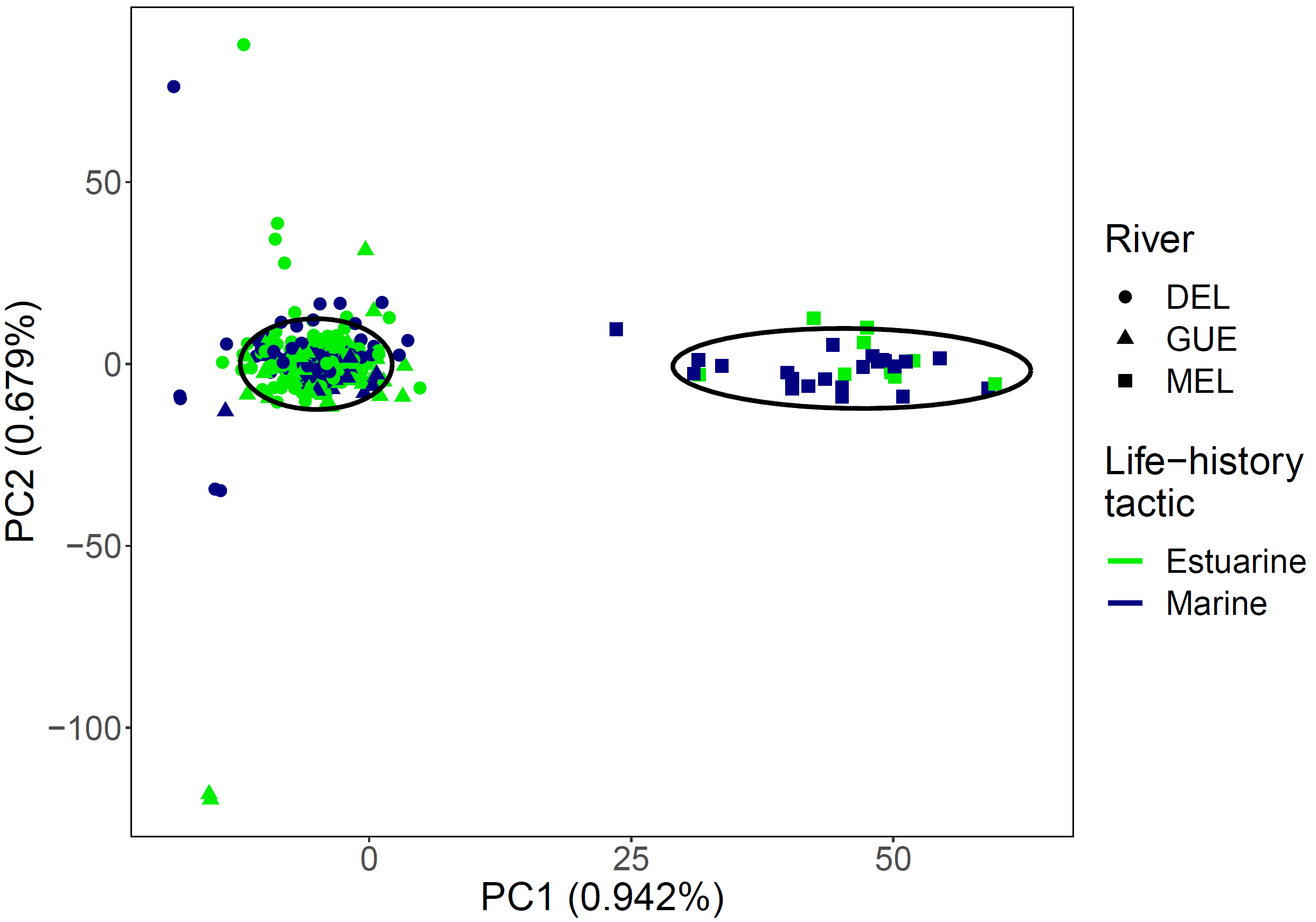

Supplement: Supplementary file 1 — Figures S1–S3. [file EVA-17-e13654-s002.docx]
