# Supplementary material for: Population genomics, life‐history tactics, and mixed‐stock subsistence fisheries in the northernmost American Atlantic salmon populations
Source: Evol Appl. 2024 Feb 22;17(2):e13654. doi: 10.1111/eva.13654 (PMC10883791; doi:10.1111/eva.13654)
Supplement: Supplementary file 2 — Tables S1–S3. [file EVA-17-e13654-s003.docx]

Supplementary Table 1 Number of individuals used for genetic structure analysis on life-history tactics. Some were captured directly in source rivers (MEL, GUE and DEL) as well as individuals captured in the Koksoak R. estuarine subsistence fisheries and subsequently assigned to these 3 rivers of origin, and classified by life-history tactic.

| **River** | **Source** | | **Mixed stock** | | **Total** |
| --- | --- | --- | --- | --- | --- |
|  | **Marine** | **Estuarine** | **Marine** | **Estuarine** |  |
| **MEL** | 20 | 7 | 2 | 3 | 32 |
| **GUE** | 7 | 23 | 22 | 47 | 99 |
| **DEL** | 11 | 31 | 54 | 77 | 173 |
| **Total** | 38 | 61 | 78 | 127 | 304 |

Supplementary Table 2 List of genes, with their biological process, identified within 20kb of the 63 SNPs significantly associated with life-history strategies identified PCadapt.

| **Chromosome** | **Gene name** | **Biological process** |
| --- | --- | --- |
| ssa02 | Eukaryotic translation initiation factor 3 subunit E | Formation of cytoplasmic translation initiation complex; translational initiation |
| ssa02 | ER membrane protein complex subunit 2 | Protein insertion into ER membrane by stop-transfer membrane-anchor sequence; tail-anchored membrane protein insertion into ER membrane |
| ssa02 | 2-oxoisovalerate dehydrogenase subunit beta, mitochondrial | Branched-chain amino acid catabolic process; cellular amino acid catabolic process; lipid metabolic process; response to camp; response to glucocorticoid; response to nutrient |
| ssa02 | Terminal nucleotidyltransferase 5A | Mrna stabilization; positive regulation of bone mineralization; positive regulation of osteoblast differentiation; regulation of ossification; response to bacterium |
| ssa02 | Inhibitor of Bruton tyrosine kinase | Negative regulation of protein phosphorylation; release of sequestered calcium ion into cytosol |
| ssa02 | Neuroendocrine convertase 1 | Neurogenesis; pancreas development; peptide biosynthetic process; peptide hormone processing; pituitary gland development; positive regulation of protein secretion; protein autoprocessing; protein processing; proteolysis; response to axon injury; response to calcium ion; response to chlorate; response to fatty acid; response to glucocorticoid; response to glucose; response to inorganic substance; response to interleukin-1; response to lipopolysaccharide; response to morphine; response to nutrient levels; response to organic cyclic compound; response to peptide hormone; response to xenobiotic stimulus |
| ssa02 | Receptor-type tyrosine-protein phosphatase U | Cell adhesion; cell differentiation; protein dephosphorylation; somite specification |
| ssa02 | Terminal nucleotidyltransferase 5A | Mrna stabilization; positive regulation of bone mineralization; positive regulation of osteoblast differentiation; regulation of ossification; response to bacterium |
| ssa02 | Heterogeneous nuclear ribonucleoprotein L-like | Mrna processing; positive regulation of RNA splicing; regulation of RNA splicing |
| ssa02 | Transmembrane protein 178A | Negative regulation of osteoclast differentiation; regulation of cytosolic calcium ion concentration |
| ssa02 | Phosphofurin acidic cluster sorting protein 2 | Apoptotic process; autophagosome assembly; endoplasmic reticulum calcium ion homeostasis; mitochondrion-endoplasmic reticulum membrane tethering; protein localization to phagophore assembly site; protein localization to plasma membrane |
| ssa02 | Signal-induced proliferation-associated 1-like protein 2 | Activation of gtpase activity; regulation of small gtpase mediated signal transduction |
| ssa02 | Signal-induced proliferation-associated 1-like protein 2 | Activation of gtpase activity; regulation of small gtpase mediated signal transduction |
| ssa02 | Egl nine homolog 1 | Cardiac muscle tissue morphogenesis; cellular iron ion homeostasis; cellular response to hypoxia; heart trabecula formation; labyrinthine layer development; negative regulation of CAMKK-AMPK signalling cascade; negative regulation of cyclic-nucleotide phosphodiesterase activity; negative regulation of DNA-binding transcription factor activity; oxygen homeostasis; peptidyl-proline hydroxylation to 4-hydroxy-L-proline; positive regulation of apoptotic process; positive regulation of neuron death; positive regulation of transcription by RNA polymerase II; regulation of angiogenesis; regulation of neuron death; regulation of postsynapse organization; regulation of protein catabolic process at postsynapse, modulating synaptic transmission; response to hypoxia; response to nitric oxide; ventricular septum morphogenesis |
| ssa02 | C-reactive protein | Acute-phase response; complement activation, classical pathway; innate immune response; negative regulation by host of viral process; regulation of interleukin-8 production |
| ssa02 | Complement C3 | Complement activation, alternative pathway; complement activation, classical pathway; defense response to bacterium; inflammatory response |
| ssa02 | Vacuolar protein sorting-associated protein 54 | Golgi to vacuole transport; homeostasis of number of cells within a tissue; lysosomal transport; musculoskeletal movement; neurofilament cytoskeleton organization; protein transport; regulation of growth; retrograde transport, endosome to Golgi |
| ssa02 | UTP--glucose-1-phosphate uridylyltransferase | F:utglucose-1-phosphate uridylyltransferase activity; brain development; glucose 1-phosphate metabolic process; glycogen biosynthetic process; glycogen metabolic process; UDP-glucose metabolic process |
| ssa02 | Malate dehydrogenase, cytoplasmic | Malate metabolic process; NAD metabolic process; NADH metabolic process; oxaloacetate metabolic process; tricarboxylic acid cycle |
| ssa02 | T-complex protein 1 subunit delta | Protein folding; response to hypoxia |
| ssa02 | tRNA pseudouridine synthase Pus10 | Primary mirna processing; trna pseudouridine synthesis |
| ssa02 | RAC-gamma serine/threonine-protein kinase | Brain morphogenesis; homeostasis of number of cells within a tissue; intracellular signal transduction; mitochondrial genome maintenance; negative regulation of cellular senescence; peptidyl-serine phosphorylation; positive regulation of angiogenesis; positive regulation of artery morphogenesis; positive regulation of blood vessel endothelial cell migration; positive regulation of cell migration involved in sprouting angiogenesis; positive regulation of cell size; positive regulation of endothelial cell proliferation; positive regulation of TOR signalling; positive regulation of vascular endothelial cell proliferation; protein phosphorylation; signal transduction |
| ssa02 | Nucleosome-remodeling factor subunit BPTF | Anterior/posterior pattern specification; brain development; chromatin remodeling; embryonic placenta development; endoderm development; negative regulation of transcription by RNA polymerase II; nucleosome mobilization; positive regulation of transcription by RNA polymerase II; regulation of transcription by RNA polymerase II; regulation of transcription, DNA-templated |
| ssa02 | Nucleolar protein 11-like | Maturation of SSU-rrna; positive regulation of transcription of nucleolar large rrna by RNA polymerase I |
| ssa02 | Cytoplasmic phosphatidylinositol transfer protein 1 | Phospholipid transport; signal transduction |
| ssa02 | Ribonuclease H2 subunit A | DNA replication, removal of RNA primer; mismatch repair; RNA catabolic process |
| ssa03 | CMRF35-like molecule 9 | Immune system process |
| ssa03 | CMRF35-like molecule 9 | Immune system process |
| ssa03 | Ras-related protein Rab-6B | Golgi organization; protein transport; vesicle-mediated transport |
| ssa04 | Polypeptide N-acetylgalactosaminyltransferase-like 6 | Protein O-linked glycosylation via threonine |
| ssa04 | N-acetylgalactosaminyltransferase 7 | Protein O-linked glycosylation |
| ssa07 | TBC1 domain family member 22B | Activation of gtpase activity |
| ssa07 | TBC1 domain family member 22A | Activation of gtpase activity |
| ssa08-29 | DnaJ homolog subfamily A member 1 | Androgen receptor signalling pathway; flagellated sperm motility; negative regulation of apoptotic process; negative regulation of establishment of protein localization to mitochondrion; negative regulation of JUN kinase activity; negative regulation of nitrosative stress-induced intrinsic apoptotic signalling pathway; negative regulation of protein ubiquitination; positive regulation of apoptotic process; protein folding; protein localization to mitochondrion; regulation of protein transport; response to heat; spermatogenesis |
| ssa08-29 | 40S ribosomal protein S6 | Glucose homeostasis; translation |
| ssa08-29 | Long-chain-fatty-acid--CoA ligase 1 | Long-chain fatty acid metabolic process; very long-chain fatty acid metabolic process |
| ssa08-29 | Guanylate cyclase soluble subunit beta-1 | Cellular response to nitric oxide; cgmp biosynthetic process; cgmp-mediated signalling; nitric oxide-cgmp-mediated signalling pathway; trans-synaptic signalling by nitric oxide, modulating synaptic transmission |
| ssa08-29 | Transmembrane protein 131-like | Negative regulation of canonical Wnt signalling pathway; negative regulation of immature T cell proliferation in thymus; Wnt signalling pathway |
| ssa08-29 | Secreted frizzled-related protein 2 | Apoptotic process; canonical Wnt signalling pathway; cell differentiation; negative regulation of canonical Wnt signalling pathway |
| ssa08-29 | Catenin alpha-2 | Axonogenesis; brain morphogenesis; cell migration; cell-cell adhesion; dendrite morphogenesis; regulation of synapse structural plasticity |
| ssa08-29 | Atrial natriuretic peptide receptor 2 | Bone development; cellular response to granulocyte macrophage colony-stimulating factor stimulus; cgmp biosynthetic process; cgmp-mediated signalling; negative regulation of meiotic cell cycle; negative regulation of oocyte maturation; ossification; positive regulation of cgmp-mediated signalling; receptor guanylyl cyclase signalling pathway; reproductive process; signal transduction |
| ssa08-29 | Atrial natriuretic peptide receptor 1 | Cell surface receptor signalling pathway; cgmp biosynthetic process; cgmp-mediated signalling; dopamine metabolic process; intracellular signal transduction; negative regulation of smooth muscle cell proliferation; positive regulation of cgmp-mediated signalling; receptor guanylyl cyclase signalling pathway; regulation of blood pressure; signal transduction |
| ssa08-29 | Transposable element Tcb1 transposase | DNA integration; transposition, DNA-mediated |
| ssa08-29 | Zinc finger protein 862 | Regulation of transcription, DNA-templated |
| ssa08-29 | Calcium-binding protein 4 | Photoreceptor cell morphogenesis; phototransduction; retinal bipolar neuron differentiation; retinal cone cell development; signal transduction; visual perception |
| ssa08-29 | PDZ and LIM domain protein 3 | Actin cytoskeleton organization; heart development; muscle structure development |
| ssa08-29 | Neuropeptide Y receptor type 2 | Adenylate cyclase-inhibiting G protein-coupled receptor signalling pathway; cardiac left ventricle morphogenesis; outflow tract morphogenesis |
| ssa08-29 | Palmitoyltransferase ZDHHC5-A | Protein palmitoylation |
| ssa08-29 | P2X purinoceptor 3 | Behavioural response to formalin induced pain; cellular response to ATP; inorganic cation transmembrane transport; neuromuscular synaptic transmission; neuronal action potential; peristalsis; positive regulation of calcium ion transport into cytosol; positive regulation of calcium-mediated signalling; positive regulation of sensory perception of pain; protein homotrimerization; regulation of synaptic plasticity; response to carbohydrate; response to cold; response to heat; response to hypoxia; response to mechanical stimulus; sensory perception of taste; signal transduction; urinary bladder smooth muscle contraction |
| ssa08-29 | Zinc finger protein 214 | Regulation of transcription by RNA polymerase II |
| ssa08-29 | Neuropilin-1a | Angiogenesis; angiogenesis involved in wound healing; animal organ morphogenesis; axon extension; axon guidance; axonal fasciculation; cartilage development; cell activation; endothelial cell chemotaxis; larval heart development; motor neuron axon guidance; neural crest cell migration; olfactory bulb axon guidance; positive regulation of cell migration involved in sprouting angiogenesis; positive regulation of filopodium assembly; regulation of angiogenesis; regulation of retinal ganglion cell axon guidance; regulation of vascular endothelial growth factor receptor signalling pathway; response to hypoxia; sprouting angiogenesis; thymus development; vascular endothelial growth factor receptor signalling pathway; vasculature development; vasculogenesis |
| ssa08-29 | Zinc finger protein 438 | Negative regulation of transcription, DNA-templated |
| ssa09 | Transcription elongation factor SPT5 | Central nervous system neuron development; hemopoiesis; negative regulation of fibroblast growth factor receptor signalling pathway; negative regulation of transcription elongation from RNA polymerase II promoter; neuron migration; positive regulation of hematopoietic progenitor cell differentiation; positive regulation of hematopoietic stem cell differentiation; positive regulation of interferon-gamma-mediated signalling pathway; positive regulation of receptor signalling pathway via JAK-STAT; positive regulation of transcription elongation from RNA polymerase II promoter; regulation of transcription by RNA polymerase II; transcription elongation from RNA polymerase II promoter |
| ssa09 | Pleckstrin homology domain-containing family G member 2 | Regulation of actin filament polymerization |
| ssa10 | Leucine-rich repeat-containing protein 15 | Negative regulation of protein localization to plasma membrane; positive regulation of cell migration; receptor-mediated virion attachment to host cell |
| ssa12 | C-C chemokine receptor type 7 | Activation of gtpase activity; calcium-mediated signalling; cell chemotaxis; cellular response to cytokine stimulus; dendritic cell chemotaxis; establishment of T cell polarity; G protein-coupled receptor signalling pathway; homeostasis of number of cells; immune response; inflammatory response; lymphocyte migration into lymph node; mature conventional dendritic cell differentiation; myeloid dendritic cell chemotaxis; negative regulation of dendritic cell apoptotic process; negative regulation of interleukin-12 production; negative thymic T cell selection; positive regulation of actin filament polymerization; positive regulation of cell adhesion; positive regulation of cell motility; positive regulation of cell-matrix adhesion; positive regulation of cytosolic calcium ion concentration; positive regulation of dendritic cell antigen processing and presentation; positive regulation of dendritic cell chemotaxis; positive regulation of ERK1 and ERK2 cascade; positive regulation of filopodium assembly; positive regulation of glycoprotein biosynthetic process involved in immunological synapse formation; positive regulation of humoral immune response; positive regulation of hypersensitivity; positive regulation of I-kappab kinase/NF-kappab signalling; positive regulation of immunological synapse formation; positive regulation of interleukin-12 production; positive regulation of JNK cascade; positive regulation of neutrophil chemotaxis; positive regulation of phosphatidylinositol 3-kinase activity; positive regulation of protein kinase activity; positive regulation of protein kinase B signalling; positive regulation of pseudopodium assembly; positive regulation of T cell costimulation; positive regulation of T cell receptor signalling pathway; regulation of dendritic cell dendrite assembly; regulation of interferon-gamma production; regulation of interleukin-1 beta production; release of sequestered calcium ion into cytosol; response to lipopolysaccharide; response to nitric oxide; response to prostaglandin E; ruffle organization |
| ssa12 | SWI/SNF-related matrix-associated actin-dependent regulator of chromatin subfamily E member 1 | Chromatin remodelling; negative regulation of transcription, DNA-templated; neurogenesis; nucleosome disassembly; positive regulation of cell differentiation; positive regulation of double-strand break repair; positive regulation of myoblast differentiation; positive regulation of stem cell population maintenance; positive regulation of T cell differentiation; regulation of G0 to G1 transition; regulation of G1/S transition of mitotic cell cycle; regulation of mitotic metaphase/anaphase transition; regulation of nucleotide-excision repair; regulation of transcription by RNA polymerase II |
| ssa12 | Insulin-like growth factor-binding protein 4 | Regulation of insulin-like growth factor receptor signalling pathway |
| ssa12 | Lysine-specific demethylase phf2 | Chromatin organization; protein demethylation |
| ssa14 | Retinoic acid receptor beta | Cell differentiation; embryonic digestive tract development; embryonic eye morphogenesis; embryonic hindlimb morphogenesis; glandular epithelial cell development; growth plate cartilage development; hormone-mediated signalling pathway; multicellular organism growth; negative regulation of cell population proliferation; negative regulation of chondrocyte differentiation; negative regulation of transcription by RNA polymerase II; neurogenesis; positive regulation of apoptotic process; positive regulation of transcription by RNA polymerase II; retinoic acid receptor signalling pathway; signal transduction; striatum development; ureteric bud development; ventricular cardiac muscle cell differentiation |
| ssa15 | Protein unc-13 homolog B} | Acrosomal vesicle exocytosis; cellular response to glucose stimulus; chemical synaptic transmission; dense core granule priming; innervation; negative regulation of synaptic plasticity; neuromuscular junction development; neuronal dense core vesicle exocytosis; phagosome maturation; positive regulation of apoptotic process; positive regulation of defense response to bacterium; positive regulation of exocytosis; positive regulation of inhibitory postsynaptic potential; positive regulation of protein secretion; positive regulation of synaptic vesicle priming; presynaptic dense core vesicle exocytosis; regulation of short-term neuronal synaptic plasticity; spontaneous neurotransmitter secretion; synaptic transmission, glutamatergic; synaptic vesicle docking; synaptic vesicle exocytosis; synaptic vesicle maturation; synaptic vesicle priming |
| ssa18 | ATP-binding cassette sub-family C member 2 | Bile acid and bile salt transport; bilirubin transport; heme catabolic process; leukotriene transport; negative regulation of gene expression; transepithelial transport; transmembrane transport; transport across blood-brain barrier; xenobiotic export; xenobiotic transmembrane transport; xenobiotic transport across blood-brain barrier |
| ssa18 | Trafficking protein particle complex subunit 14 | Cilium assembly; regulation of cell population proliferation |
| ssa18 | Metallo-beta-lactamase domain-containing protein 1 | Histone mrna metabolic process; positive regulation of G1/S transition of mitotic cell cycle |
| ssa20 | Arf-GAP domain and FG repeat-containing protein 1 | Cell differentiation; mrna export from nucleus; spermatogenesis |
| ssa21 | DnaJ homolog subfamily C member 15 | Cellular response to starvation; negative regulation of mitochondrial electron transport, NADH to ubiquinone; negative regulation of protein-containing complex assembly; protein import into mitochondrial matrix; regulation of lipid metabolic process |

Supplementary Table 3 Number of individuals captured in the Koksoak R. estuarine subsistence fisheries subsequently assigned to their river of origin and classified by life-history tactic.

| **Year** | **Marine** | | | | **Estuarine** | | | | **Total** |
| --- | --- | --- | --- | --- | --- | --- | --- | --- | --- |
|  | **MEL** | **GUE** | **DEL** | **CAN** | **MEL** | **GUE** | **DEL** | **CAN** |  |
| **2020** | 0 | 10 | 27 | 0 | 3 | 31 | 59 | 11 | 141 |
| **2021** | 2 | 12 | 27 | 0 | 0 | 16 | 18 | 2 | 77 |
| **Total** | 2 | 22 | 54 | 0 | 3 | 47 | 77 | 13 | 218 |

It should be noted that some individuals have not been included in this table because their tactics could not be identified, or because they have been identified as mixed, i.e. they have adopted both estuarine and marine migratory behavior. Generally speaking, these fish remain in the estuary during the first summer following smoltification, but migrate to the sea during the second summer following smoltification. Of the individuals whose tactics could not be identified, 2 were assigned to GUE R. in 2020. In 2021, 2 individuals were assigned to MEL R., 7 to GUE R., 13 to DEL R. and 1 to CAN R. whose tactics could not be identified. Among the individuals identified as having adopted a mixed tactic and caught in 2020, 3 were assigned to GUE R. and 5 to DEL R. As for the 2021 catches, 1, 9 and 16 individuals respectively were assigned to MEL R., GUE R. and DEL R., and had adopted this tactic. Considering these samples, the total number of individuals captured in 2020 is 151 and 126 in 2021, with an overall total of 277. Finally, the difference of 3 individuals compared to the Table 1, is due to the removal of 3 individuals that could not be assigned to their river of origin.
