# Supplementary material for: Population genomics, life‐history tactics, and mixed‐stock subsistence fisheries in the northernmost American Atlantic salmon populations
Source: Evol Appl. 2024 Feb 22;17(2):e13654. doi: 10.1111/eva.13654 (PMC10883791; doi:10.1111/eva.13654)
Supplement: Supplementary file 3 — Data S1. [file EVA-17-e13654-s001.docx]

*Life-history tactic identification using scales*

The identification of migratory tactic was done by Ministère de l’Environnement, de la Lutte aux Changements Climatiques, de la Faune et des Parcs (MELCCFP) technicians with over 10 years of experience in Atlantic salmon scale reading. Scales were mounted between microscopes slides, and then visualized with a stereo microscope according to the method of Côté et al. (1984) and Robitaille et al. (1986). Individuals caught within the Koksoak R. (source rivers as well as estuarine fishery) have been separately analysed by three technicians to ensure consistency and maximize correct assessment. Fish caught within FEU, BAL, GEO and NST have not been subjected to this scale analyses. Since scale growth is proportional to the fish's growth and is largely dependent on the environment in which it is found, the scales allow us to assess growth patterns and ultimately to identify the migration tactic. In simple terms, a scale is made up of growth rings (or circuli) that accumulate as the fish grows, with the distance between each ring, linked to growth rate, varying according to environmental conditions. Thus, for a given environment, the circuli should be further apart when conditions are favorable for growth, but closer together when conditions are less favorable (e.g. in summer compared to winter or an unproductive habitat). This method can be used to determine an individual's age by counting the number of winter marks on a scale (designated as circuli so close to one another that several circuli form a large dark band) (ICES, 2011), but can also be used to compare growth rates in different environments, such as river, estuary and the sea. Applying this theory and considering that landlocked salmon spend their entire life in freshwater, a relatively poor environment compared to the estuary or the sea, they are characterized by fairly slow and constant growth. This freshwater environment results in scales with circuli that are consistently close together over the years, suggesting that the individual did not migrate from freshwater to a more productive environment (Figure 1). For estuarine and marine salmon, the growth pattern prior to smoltification (years before the first migration to the estuary/sea), as well as the first months of the first summer in the feeding area, is similar. The difference in growth that allows us to distinguish between these 2 life-history tactics takes place in late fall and winter. Marine salmon are at sea during the fall and winter, and are exposed to a relatively gradual reduction in water temperature. In contrast, estuarine salmon are known to migrate from the estuary to freshwater during the fall and to overwinter in river (Robitaille et al. 1986). Compared to the temperature reduction at sea, the temperature reduction in the river is relatively drastic, and so is the growth of the salmon. This difference in conditions between marine and estuarine salmon results in different circuli signatures that can be observed on the scales. Indeed, considering a scale as a timeline and reading it from the first year post smoltification (Figure 1) to the scale edge (corresponding to when the scale was sampled), circuli of a marine salmon are, for each year at sea, relatively far apart during summers and become gradually closer together as the winter mark approaches. In estuarine salmon, circuli are also, for each year of estuarine growth, relatively far apart during summers, but become drastically closer together compared to marine salmon as they approach the winter mark (Figure 1). This method has no predefined threshold in terms of the number of circuli or the distance between them. However, the environments to which each tactic is exposed are so different that it is possible to differentiate them visually. Moreover, although microchemical analyses of scales or otoliths would have allowed us to use a more specific threshold in the identification of these tactics, scale reading (as described above) is more cost effective and less time consuming. Moreover, this method has been used for many years to differentiate between salmon life-history tactics in Ungava Bay, and its effectiveness has recently been confirmed by microchemical analyses of otoliths (Brûlé, 2022).

Figure 1 Modified from Figure 4 of Brûlé (2022); representation of the circuli (i.e., growth) of salmon growing A) in freshwater, B) in estuary and C) at sea. Specimens were captured in the Koksoak R.


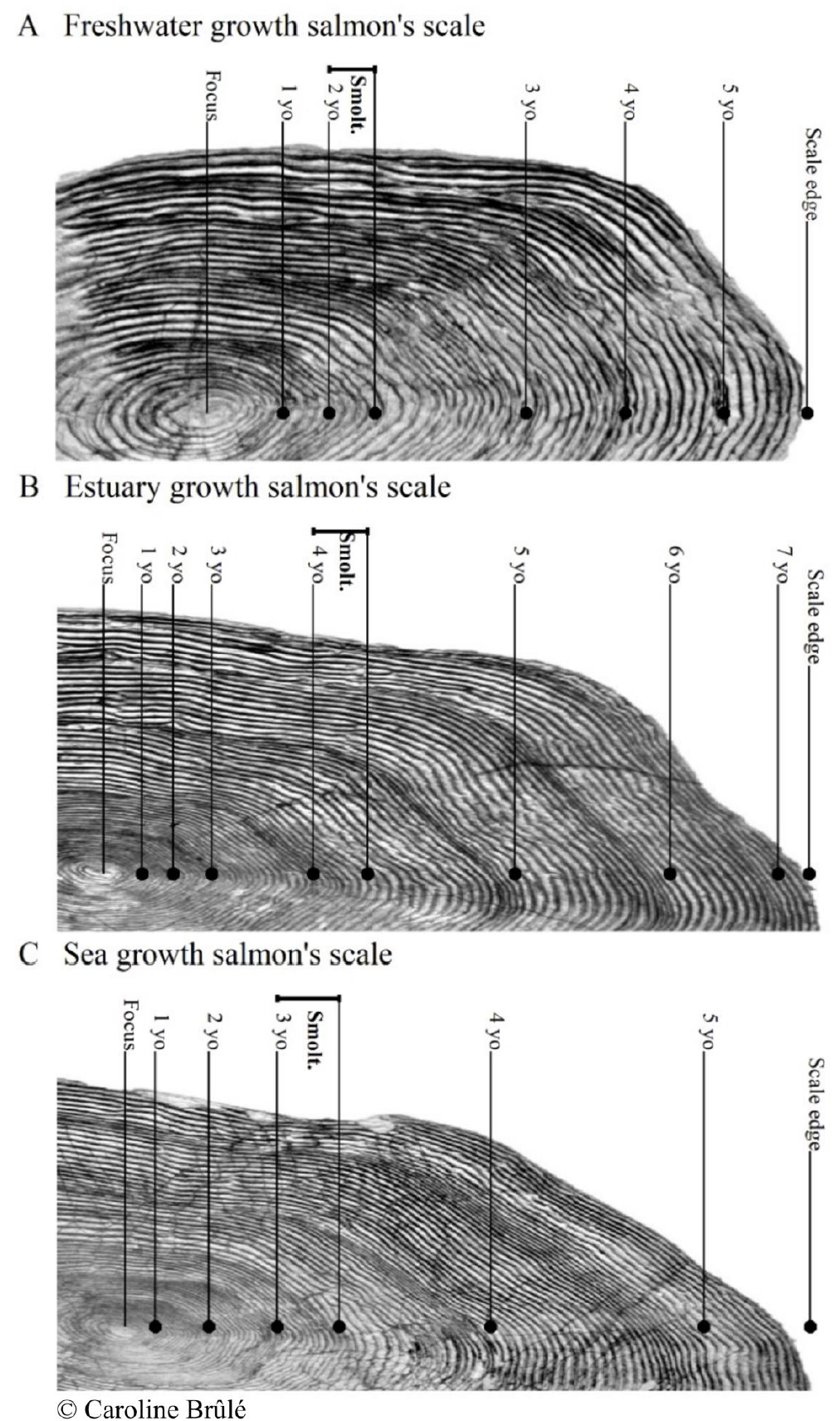


**Literature cited**

Brûlé, C. (2022). Occurrence de saumons atlantique (*Salmo salar*) estuariens dans la région de la baie d'Ungava (Nunavik, QC) confirmée par la chimie des otolithes et la morphologie des écailles. Mémoire de maîtrise, Université du Québec à Chicoutimi. 80p.

Côté, Y., Babos, I., and Robitaille, J.A. (1984). Caractéristiques scalimétriques des saumons du Koksoak (Ungava, Québec). *Naturaliste canadien*, 111: 401-409.

ICES. (2011). Report of the Workshop on Age Determination of Salmon (WKADS). *ICES Document CM 2011/ACOM:44 67pp*, 18‐20

Robitaille, J.A., Côté, Y., Shooner, G., Hayeur, G. (1986). Growth and maturation patterns of Atlantic salmon, *Salmo salar*, in the Koksoak River, Ungava, Quebec. *Canadian Special Publication of Fisheries and Aquatic Sciences*, 89: 62-69.
